# Novel halotolerant fungal endophytes from hypersaline coastal ecosystems as sustainable biostimulants for early growth performance of tomato and wheat under saline stress

**DOI:** 10.3389/fpls.2026.1905810

**Published:** 2026-08-18

**Authors:** Victoria Huertas, Fernando Diánez, Carmen Palazón, Melanie D. Gómez-Herrera, María Teresa Planas, Mila Santos

**Affiliations:** 1 Departamento de Agronomía, Escuela Superior de Ingeniería, Universidad de Almería, Almería, Spain; 2 Departamento de Tecnología del Medio Ambiente, Instituto Universitario de Investigación Marina (INMAR) Universidad de Cádiz, Puerto Real (Cádiz), Spain; 3 Laboratorio de Fitopatología, Facultad de Ciencias Agrarias, Universidad Nacional del Nordeste, Corrientes, Argentina

**Keywords:** abiotic stress tolerance, dark septate endophytes, halotolerance, plant growth-promoting fungi, redox homeostasis, sustainable agriculture, Trichoderma

## Abstract

Soil salinization severely restricts seed germination, seedling establishment, and vegetative growth in key crops such as tomato (*Solanum lycopersicum* L.) and wheat (*Triticum aestivum* L.). Endophytic fungi from extreme environments are promising biostimulants to alleviate salinity stress, yet systematic assessments of isolates from hypersaline coastal ecosystems remain scarce. Here, we evaluated 28 endophytic fungal isolates from saline coastal ecosystems of the southeastern Iberian Peninsula using a four-stage pipeline: (i) *in vitro* characterization of salinity and temperature tolerance, (ii) germination bioassays under increasing NaCl gradients, (iii) seedling development under saline stress in controlled-environment pot experiments, and (iv) antioxidant enzyme profiling (POD, CAT, SOD) in both crops. *In vitro* screening revealed broad phenotypic diversity driven by a significant isolate × temperature × NaCl interaction, resolving two functional superclusters: a thermotolerant group led by *Sordaria fimicola* (BC05, BC06) with generalist thermal plasticity, and a stress-sensitive mesophilic group including several *Trichoderma* isolates, with BC47 (*Nothophoma gossypiicola*) emerging as a halotolerant specialist. Fungal inoculation significantly modulated germination, with responses strongly dependent on crop species, salinity level, and endophyte identity. In tomato, isolates BC06 (*S. fimicola)*, BC17 (*Clarireedia* sp.), BC47, and BC52 (*N. gossypiicola*) maintained radicle emergence at 100 mM NaCl, whereas in wheat BC06 was the only isolate capable of overcoming complete germination inhibition at 300 mM NaCl, markedly extending the osmotic tolerance limit of the species. Under saline pot conditions, inoculation enhanced tomato leaf area by up to 60% and root dry weight by up to 340%, while wheat responses were mostly neutral or negative, with BC24 (*Trichoderma gamsii)* as the sole shoot biomass enhancer (+58.1%) and BC50 (*T. saturnisporum*) uniquely improving the Dickson Quality Index. Antioxidant enzyme activities displayed contrasting, host- and context-specific patterns between crops. Overall, this study provides the first evidence of *S. fimicola, Clarireedia narcissi*, and *N. gossypiicola* as effective fungal biostimulants under saline stress and highlights the biotechnological potential of non-conventional endophytes from extreme coastal environments as a basis for the rational selection of sustainable bioinoculants for salt-affected agricultural systems.

## Graphical Abstract

**Figure:**
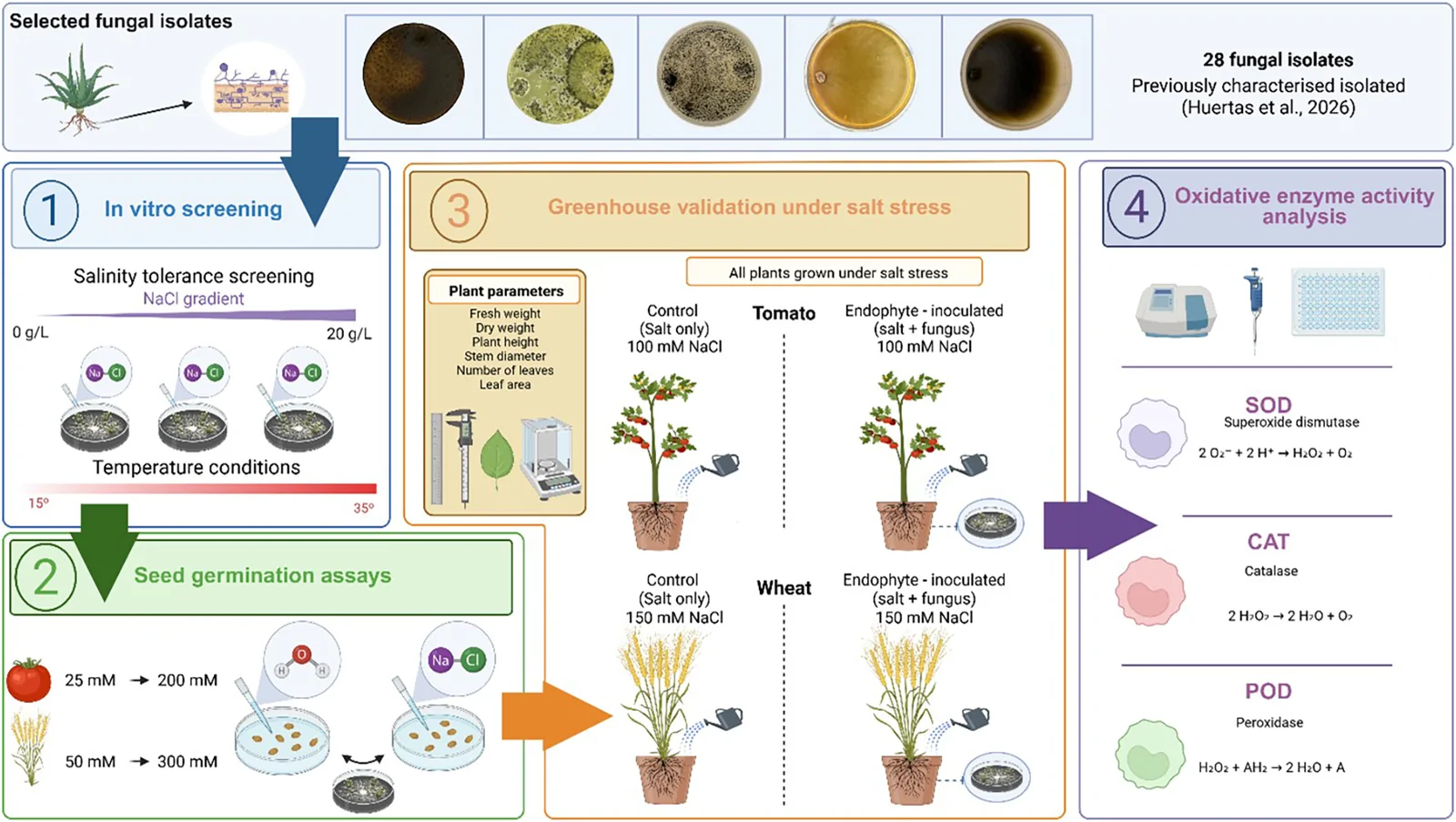

## 1 Introduction

Soil salinization represents one of the major threats to global agriculture and food security, particularly in arid and semi-arid regions, where it reduces arable land and limits the yield of numerous staple crops (Hassani et al., 2021; Machado and Serralheiro, 2017; Balasubramaniam et al., 2023). Currently, salinity affects hundreds of millions of hectares of agricultural land and can lead to yield losses of 20–50% across various production systems. Furthermore, its impact is projected to increase under climate change scenarios, rising sea levels, and inadequate irrigation and drainage management (Navarro-Torre et al., 2023; Tarolli et al., 2024). At the physiological and edaphic levels, salt stress compromises water and nutrient uptake, disrupts ionic homeostasis, deteriorates soil structure, and modifies microbial diversity, with direct consequences for the sustainability of agricultural production and the Sustainable Development Goals (SDGs) (Morales-Vargas et al., 2024).

Conventional strategies to mitigate salinity, such as breeding for tolerant crops, optimizing irrigation and drainage, or applying soil amendments, have shown variable efficacy and may present economic, technical, or environmental limitations when applied in isolation (Mohanavelu et al., 2021; Mukhopadhyay et al., 2020). In this context, bio-strategies based on beneficial soil microorganisms have received increasing attention due to their ability to enhance plant tolerance to abiotic stress. Among these, fungal endophytes represent a promising alternative, as they colonize internal plant tissues without causing apparent disease symptoms and can modulate systemic responses related to growth, nutrition, and stress tolerance (Kumar et al., 2025; Gupta et al., 2021).

Several studies have demonstrated that fungal endophytes can enhance plant salt tolerance through mechanisms such as osmotic adjustment, hormonal regulation, reinforcement of the antioxidant system, improved nutrient uptake, and the maintenance of ion homeostasis (Fontana et al., 2021; Zou et al., 2023; Gowtham et al., 2024). These effects are particularly relevant under moderate to high salinity conditions, where the activation of metabolic and defensive pathways plays a key role in plant survival and productivity (Fontana et al., 2021; Gowtham et al., 2024). Therefore, the use of fungal endophytes as biostimulants constitutes a sustainable biotechnological strategy to reduce the negative effects of salinity in agricultural systems.

Among fungal endophytes, the genus *Trichoderma* stands out for its cosmopolitan distribution, its history as a biocontrol agent, and its efficacy as a biostimulant across a wide range of crops (Hu et al., 2025). *Trichoderma* colonizes internal plant tissues without causing harm, establishing a mutualistic symbiosis that translates into direct and indirect benefits for the host, particularly under saline conditions (Verma et al., 2022). Various studies show that, under salt stress, the plant–*Trichoderma* association consistently increases shoot and root biomass, chlorophyll content, and antioxidant enzyme activity, reducing oxidative stress markers, including superoxide radicals and lipid peroxidation products, and enhancing the enzymatic detoxification of oxidative damage (Morales-Vargas et al., 2024; Gupta et al., 2021; Yasmeen and Siddiqui, 2017). For instance, in cereals, inoculation with *Trichoderma harzianum* or *T. longibrachiatum* markedly improved the growth of wheat, barley, and maize in the presence of NaCl. In wheat, *Trichoderma* isolates increased net photosynthesis, water-use efficiency, and biomass under 150–200 mM NaCl (Santos et al., 2025; Zhang et al., 2016). In maize, co-inoculation with arbuscular mycorrhizal fungi and *T. harzianum* increased root dry weight, root volume, and total biomass under salinity, accompanied by higher activity of superoxide dismutase (SOD), catalase (CAT), and ascorbate peroxidase (APX), as well as the accumulation of protective metabolites (Oljira et al., 2020). Similarly, in tomato, endophytic colonization by *T. asperellum*, *T. viride*, *T. guizhouense*, and *T. harzianum* mitigates salinity by improving germination, seedling survival, growth, and chlorophyll content under 50–200 mM NaCl (Rubio et al., 2017; Bader et al., 2020). In Cucurbitaceae, such as cucumber and zucchini, salt-tolerant strains of *T. harzianum*, *T. viride*, and *T. atroviride* attenuate the growth decline induced by 50–200 mM NaCl while simultaneously reducing the severity of root diseases caused by *Fusarium oxysporum* (Zhang et al., 2025). These effects are associated with the integration of various mechanisms, including antioxidant reinforcement, osmotic adjustment, photosynthetic and nutritional enhancement, and the maintenance of ion homeostasis (Eftekhari et al., 2025; Konappa et al., 2023; Hu et al., 2025).

On the other hand, Dark Septate Endophytes (DSE) constitute a diverse group of ascomycetous fungi, characterized by melanized hyphae and microsclerotia. They colonize more than 600 plant species without marked host specificity and are particularly frequent and abundant in saline, arid, and desert environments (Santos et al., 2021; Mei et al., 2025). In various crops and woody species (e.g., tomato, rice, legumes, and desert shrubs), DSE inoculation has been reported to increase shoot and root biomass, improve germination and chlorophyll content, and enhance nutritional status, particularly with respect to nitrogen and phosphorus acquisition, even under conditions of limited nutrient availability (Santos et al., 2016; Hou et al., 2021a; Gaber et al., 2023; Huertas et al., 2024; Lalthazuali et al., 2025). Under stress conditions, DSE-associated plants exhibit a reinforced antioxidant system and osmotic adjustment; modification of root architecture with increased soil exploration; changes in the rhizospheric microbiome, favoring arbuscular mycorrhizal fungi and growth-promoting bacteria; and a modulation of ionic balance, particularly the K⁺/Na⁺ ratio, whose effect can be positive or neutral depending on the DSE species, host, and salinity level (He et al., 2019; Huertas et al., 2024). Comparisons between *Trichoderma* and DSE reveal that, although both fungal groups can improve plant salt tolerance, their mechanisms and effects tend to differ and, occasionally, complement each other (Fu et al., 2021; He et al., 2022a; He et al., 2022b; Saha et al., 2024). Despite the solid foundation for using *Trichoderma* and DSE as biostimulants under salinity, systematic comparative studies evaluating both fungal groups under standardized experimental conditions are still required, as is the functional characterization of new isolates from underexplored environments as potential biostimulants, which constitutes the primary motivation of the present study (Irshad et al., 2023; Huertas et al., 2024; Eftekhari et al., 2025).

Therefore, the general objective of this study was to evaluate the biostimulant potential of 28 fungal endophytic isolates, isolated from saline coastal ecosystems in the southeastern Iberian Peninsula, on the germination and early development of *Solanum lycopersicum* L. and *Triticum aestivum* L. under salt stress. To achieve this, the study integrated: (i) the *in vitro* characterization of the isolates’ salinity tolerance under different thermal regimes and NaCl concentrations; (ii) the evaluation of the effect of fungal inoculation on alleviating salt-induced germination inhibition across increasing salinity gradients, identifying their capacity to broaden the osmotic tolerance range in both crops; (iii) the quantification of fungal inoculation efficacy in promoting vigor and vegetative development under persistent salinity, analyzing the isolates’ ability to mitigate growth limitations imposed by salt stress; and (iv) the analysis of POD, CAT, and SOD enzyme modulation to establish the relationship between the symbiont-induced antioxidant response and the mitigation of abiotic stress in the selected crops.

## 2 Materials and methods

### 2.1 Fungal isolates

A total of 28 endophytic fungal isolates were used in this study, obtained from the roots of native plants sampled between February and July 2023 across coastal ecosystems of the southeastern Iberian Peninsula. Collection sites included salt marshes, Cabo de Gata-Níjar Natural Park, the Adra lagoons, and the wetlands of Roquetas de Mar. These locations were selected based on their extreme environmental conditions, characterized by arid and semi-arid climates, pronounced water deficit, and elevated soil salinity — conditions recognized to drive the development of adaptive tolerance mechanisms in the associated microbiota (Huertas et al., 2026).

The isolation, molecular identification and selection of this fungal collection were described in detail in our previous companion study (Huertas et al., 2026) and are summarized here. Endophytes were recovered from surface-sterilized root segments following a modification of the method of Surono and Narisawa (2017), and plated on PDA amended with chloramphenicol (100 µg mL^–1^), yielding 415 isolates from 68 root samples. Based on colony morphology and growth pattern on solid media, 52 isolates were retained, prioritizing those with high sporulation capacity or producing microsclerotia typical of dark septate endophytes (DSEs), since propagule production is a key requirement for the subsequent development of stable bioformulations. Of these, 50 were identified molecularly by ITS sequencing (primers ITS1/ITS4) followed by BLAST comparison against the NCBI GenBank database, and all sequences were deposited in GenBank; accession numbers, closest related species and sequence identity values are reported in the companion paper. The 28 isolates evaluated in the present work correspond to the subset that combined the strongest *in vitro* antagonism against phytopathogens with plant growth-promoting traits (IAA and siderophore production, phosphate or potassium solubilization) and the absence of pathogenicity on tomato, pepper and bean seedlings (**Table 1**). Prior to experimental use, isolates were reactivated by culturing on Potato Dextrose Agar (PDA; 200 g potato infusion, 20 g dextrose, and 17 g agar per liter) and incubated at 25 °C for seven days.

**Table 1:** Origin and taxonomic identification of fungal isolates recovered from the roots of native plants in coastal ecosystems of the southeastern Iberian Peninsula.

Isolate	Closely related species	Host plant	GenBank No
BC01	*Torula* sp.	–*	PV664294
BC04	*Macrophomina phaseolina*	*–*	PV664359
BC05	*Sordaria fimicola*	*Hyparrhenia hirta*	PV664364
BC06	*Sordaria fimicola*	*Hyparrhenia hirta*	PV664365
BC08	*Macrophomina phaseolina*	*Asteriscus aquaticus*	PV664381
BC11	*Torula* sp.	*–*	PV664384
BC12	*Trichoderma gamsii*	*Tymelaea hirsuta*	PV664385
BC13	*Torula* sp.	*–*	PV664386
BC17	*Clarireedia* sp.	*Sedum sediforme*	PV664388
BC18	*Clarireedia* calopus	*Sedum sediforme*	PV664389
BC20	*Trichoderma gamsii*	*Genista umbellata*	PV664391
BC24	*Trichoderma gamsii*	*Genista umbellata*	PV664395
BC25	*Macrophomina phaseolina*	*–*	PV664396
BC27	*Clarireedia narcissi*	*Genista umbellata*	PV664398
BC30	*Clarireedia narcissi*	*Genista umbellata*	PV664401
BC31	*Didymella longicolla*	*Genista umbellata*	PV698399
BC34	*Clarireedia narcissi*	*Genista umbellata*	PV698401
BC37	*Nothophoma quercina*	*Andryala ragusina*	PV698403
BC42	*Phoma* sp.	*Marrubium alysson*	PV698408
BC44	*Nothophoma anigozanthi*	*Marrubium alysson*	PV698410
BC45	*Nothophoma gossypiicola*	*Artemisia barrelieri*	PV698411
BC46	*Nothophoma gossypiicola*	*Anthyllis cytisoides*	PV698412
BC47	*Nothophoma gossypiicola*	*Anthyllis cytisoides*	PV698413
BC48	*Trichoderma saturnisporum*	*Anthyllis cytisoides*	PV698414
BC49	*Trichoderma saturnisporum*	*Anthyllis cytisoides*	PV698415
BC50	*Trichoderma saturnisporum*	*Anthyllis cytisoides*	PV698416
BC51	*Phoma* sp.	*–*	PV698417
BC52	*Nothophoma gossypiicola*	*Salsola oppositifolia*	PV698418

*A dash (−) indicates that the host plant could not be identified at the time of collection. For each isolate, the associated host plant and the closest related species, determined by ITS sequence analysis, are provided along with their GenBank accession numbers.

### 2.2 Assessment of fungal growth under salinity stress and temperature gradients

The growth capacity of selected isolates under salinity stress was evaluated through *in vitro* assays. Isolates were cultivated on PDA supplemented with NaCl at concentrations of 0, 2, 5, 10, 15, and 20 g L^–1^. For each treatment, a 5-mm mycelial plug excised from the actively growing margin of a colony was centrally placed on a Petri dish containing the corresponding medium. Five replicates were established per isolate, NaCl concentration, and temperature regime (28 isolates × 6 NaCl levels × 3 temperatures × 5 replicates), resulting in a total of 90 Petri dishes per isolate. Colony radius was recorded daily during incubation in darkness at three temperature regimes: 15, 25, and 35 °C.

Salt tolerance was quantified using the mean daily radial growth rate (GR), defined as the average increase in colony radius between consecutive observations normalized by the elapsed time interval, and calculated as follows: GR= d_2_- d_1_/t_2_-t_1_, where d_2_ and d_1_ represent the colony radius (cm) at the final and initial observations, respectively, and t_2_ − t_1_ denotes the time interval in days.

To characterize the phenotypic diversity and physiological plasticity of the isolates under abiotic stress, hierarchical cluster analysis (HCA) was performed using the ClustVis web tool. Radial growth data were normalized by Z-score transformation (global scaling) to enable cross-condition comparisons across all experimental treatments. Clustering was applied to both rows (temperature and salinity treatments) and columns (fungal isolates) using Euclidean distance as the dissimilarity metric and the Unweighted Pair Group Method with Arithmetic Mean (UPGMA) as the linkage algorithm. This configuration enabled the identification of functional clusters based on global growth profiles, minimizing the bias introduced by differences in absolute growth rates between optimal and restrictive conditions. The resulting heatmap was exported in vector format to ensure high-resolution visualization for the interpretation of functional clusters. Principal Component Analysis (PCA) was additionally performed on the same normalized dataset to identify the main axes of variation among isolates in response to combined thermal and osmotic stress.

### 2.3 Germination assays: effect of salinity stress and fungal inoculation

The effect of salinity on the germination of *Solanum lycopersicum* and *Triticum aestivum* was evaluated through *in vitro* assays conducted under controlled conditions. Seeds were subjected to species-specific surface sterilization protocols prior to use. *S. lycopersicum* seeds were immersed in 1% sodium hypochlorite (NaClO) for 5 min, followed by three sequential rinses with sterile distilled water (30, 15, and 5 min, respectively) to eliminate disinfectant residues. *T. aestivum* seeds were disinfected sequentially in 1% NaClO (2 min), 70% ethanol (2 min), and 30% ethanol (1 min), rinsed with sterile distilled water (5 min), and then imbibed in distilled water for 12 h (Schulz et al., 1993). All seeds were subsequently air-dried on sterile filter paper under a laminar flow hood.

Surface-sterilized seeds were transferred to square germination plates (120 × 120 mm) lined with sterile filter paper at a density of 12 seeds per plate for *S. lycopersicum* and 16 seeds per plate for *T. aestivum*. Salinity stress was imposed by applying increasing NaCl concentrations: 25, 50, 75, and 100 mM for *S. lycopersicum*, and 50, 75, 100, 150, 200, 250, and 300 mM for *T. aestivum*; distilled water served as the control treatment in both cases (Prajapati et al., 2024). Each of the 28 fungal isolates was evaluated in triplicate per treatment, with each replicate consisting of one germination plate containing 12 seeds (*S. lycopersicum*) or 16 seeds (*T. aestivum*), respectively (n = 3 plates × 12–16 seeds per NaCl concentration and isolate). Plates were moistened with 3 mL of sterile water, and each seed was individually inoculated with 30 µL of a fungal suspension adjusted to 10^6^ colony-forming units (CFU) mL^–1^, or with six microsclerotia, depending on the morphological characteristics of the isolate. Plates were incubated in darkness at 25 °C, and germination was monitored daily throughout the experimental period. The germination rate per plate was recorded to determine the limiting NaCl concentration for each plant species. Germination was defined as radicle emergence reaching a minimum length of 2 mm.

### 2.4 Pot experiments under salinity stress

Tomato (*Solanum lycopersicum* L.) plants were grown in 300 mL pots filled with non-sterilized commercial peat-based substrate to approximate realistic horticultural conditions. Wheat (*Triticum aestivum* L.) plants were grown in 12 × 12 × 14 cm plastic pots containing vermiculite to facilitate root recovery and cleaning. All pots were fitted with drainage holes and placed on collection trays to prevent cross-contamination between treatments.

For fungal inoculation, each pot substrate was pre-inoculated with three 5-mm mycelial discs excised from the actively growing margin of fungal colonies maintained on PDA. Discs were inserted at approximately 3–4 cm depth and evenly distributed within the root zone. Inoculated pots were maintained under growth chamber conditions (25 ± 2 °C) for 10 days to allow fungal establishment prior to sowing. Control pots received three sterile PDA discs in place of fungal inoculum. Pre-germinated seeds (under non-saline conditions, 0 mM NaCl) individually inoculated with the corresponding fungal isolate were used for sowing. Two seedlings were transplanted per pot and, following establishment, thinned to one per pot, retaining the most uniform individual. The experiment followed a completely randomized design with 20 replicates per treatment. Plants were maintained in a growth chamber at 25 ± 2 °C under a 16 h light/8 h dark photoperiod and 60–70% relative humidity.

Salinity stress was imposed by irrigating with NaCl solutions at concentrations selected based on prior *in vitro* germination assays (Rodriguez et al., 2008): 100 mM for tomato and 150 mM for wheat. Salt treatment was initiated at 15 days after sowing (DAS) for tomato and 9 DAS for wheat. Plants were irrigated every two days with 100 mL pot^–1^ for tomato and 50 mL pot^–1^ for wheat, maintaining substrate moisture near field capacity to minimize osmotic fluctuations. Plants were harvested after 42 days of salt treatment for tomato and 58 days for wheat. Growth parameters assessed included plant height, leaf number, and fresh and dry biomass of shoot and root systems. For biomass determination, shoots and roots were separated, immediately weighed to record fresh mass, and subsequently oven-dried at 65 °C for 72 h until constant weight was reached.

For tomato, leaf area (LA) was quantified by photographing fully expanded leaves against a white background with a known-area reference marker. Images were processed in ImageJ (v. 1.53) by converting to 8-bit grayscale and applying a threshold for leaf tissue segmentation; LA (cm²) was subsequently calculated based on pixel density (Zhu, 2001).

Root colonization by the inoculated fungal strains was confirmed by (i) re-isolation of surface-sterilized root fragments on selective culture medium, and (ii) microscopic observation of root sections stained with ink-vinegar (Vierheilig et al., 1998). Briefly, roots were cleared in 10% KOH (90–95 °C, 10–15 min), stained in 5% black ink in white vinegar (90 °C, 3–5 min), destained in acidified water, and examined under a light microscope (40–100×) to confirm fungal structures within the root cortex.

### 2.5 Antioxidant enzyme activities

Antioxidant enzyme activities, peroxidase (POD), catalase (CAT), and superoxide dismutase (SOD), were determined from extracts obtained from dry plant material using three independent biological replicates per isolate and treatment (n = 3). Plant tissues were homogenized at a 1:3 ratio (w/v) in 50 mM potassium phosphate buffer (pH 7.2) supplemented with 5 mM dithiothreitol (DTT), 1 mM EDTA, and 2% (w/v) polyvinylpyrrolidone (PVP) (Zou et al., 2023). The homogenate was centrifuged at 11,000 × g for 10 min at 4 °C, and the resulting supernatant was collected for enzyme activity measurements (Fimognari et al., 2020).

POD activity was measured spectrophotometrically using the guaiacol oxidation assay, based on the formation of tetraguaiacol. The reaction mixture (3 mL total volume) consisted of 50 mM sodium phosphate buffer (pH 7.0), 3.33 mM guaiacol, 4 mM H_2_O_2_, and 20 µL of enzyme extract, and was carried out at 25–28 °C. The increase in absorbance at 470 nm was recorded every 30 s over 3 min. POD activity was calculated using the molar extinction coefficient of tetraguaiacol (ϵ = 26.6 mM^–1^ cm^–1^) (De Almeida et al., 2019) and expressed as enzyme activity units per 100 g of dry weight (U·100 g^–1^ DW), where one unit (U) is defined as the amount of enzyme required to catalyze the formation of 1 μmol of tetraguaiacol per minute under the assay conditions.

CAT activity was determined spectrophotometrically by monitoring H_2_O_2_ decomposition at 240 nm, using the same supernatant obtained for POD determination (Anjum et al., 2016). The reaction mixture (3.0 mL final volume) contained 50 mM potassium phosphate buffer (pH 7.0) and an appropriate volume of enzyme extract. The reaction was initiated by adding H_2_O_2_ to a final concentration of 10–30 mM, a range selected to prevent catalase inactivation and bubble formation during measurement. The decrease in absorbance at 240 nm was recorded every 15–30 s over 2–3 min at 25–28 °C (Hadwan et al., 2024). Catalase (CAT) activity was calculated from the linear portion of the H_2_O_2_ decay curve using the molar extinction coefficient of H_2_O_2_ (ϵ = 39.4 M^–1^ cm^–1^) (Farman and Hadwan, 2021) and expressed as enzyme activity units per gram of dry weight (U g^–1^ DW), where one unit (U) is defined as the amount of enzyme required to catalyze the decomposition of 1 μmol of H_2_O_2_ per minute under the assay conditions.

SOD activity was determined based on the inhibition of photochemical nitro blue tetrazolium (NBT) reduction, following the method of Beauchamp and Fridovich (1971) adapted to a 96-well microplate format. Dry tissue was homogenized in cold 50 mM potassium phosphate buffer (pH 7.8) and centrifuged at 4,000 × g for 15 min at 4 °C; the supernatant was used immediately. Each well (200 µL final volume) contained 50 mM sodium phosphate buffer (pH 7.8), 13 mM L-methionine, 75 µM NBT, 0.1 mM EDTA, 2 µM riboflavin, and enzyme extract. The reaction was initiated by adding riboflavin and exposing plates to uniform white light (~150 µmol m⁻² s^–1^) for 30 min at room temperature; duplicate wells kept in darkness served as blanks. Absorbance was subsequently read at 560 nm to quantify NBT-formazan formation. SOD activity was expressed as enzyme activity units per gram of dry weight (U g^–1^ DW), where one unit (U) is defined as the amount of enzyme extract that inhibits 50% of the photochemical reduction of nitro blue tetrazolium (NBT) to formazan under the assay conditions. Spearman rank correlation coefficients were calculated to evaluate relationships between antioxidant enzyme activities (POD, CAT, SOD) and plant biomass parameters (PSA, PSR, and total biomass) across the 28 fungal strains, using the corrplot package in R.

### 2.6 Statistical analysis

All experimental data were analyzed using two-way analysis of variance (two-way ANOVA) to assess the main effects of fungal inoculation (I) and salinity level (S), as well as their interaction (I × S). Prior to analysis, normality and homoscedasticity were verified using the Shapiro–Wilk and Levene’s tests, respectively. When significant differences were detected, Tukey’s Honestly Significant Difference (HSD) *post-hoc* test was applied for pairwise comparisons (P < 0.05). Hierarchical cluster analysis (HCA) and Principal Component Analysis (PCA) were used to characterize phenotypic diversity in fungal growth responses (Section 2.2), while Spearman rank correlation was applied to assess associations between antioxidant enzyme activities and biomass parameters (Section 2.5). Data processing and ANOVA were conducted in Statgraphics (Statgraphics Technologies Inc., The Plains, VA, USA); HCA and PCA were performed in ClustVis. The Dickson Quality Index (DQI) was calculated using custom R scripts (v. 4.2.0; R Core Team, 2022) integrating biomass and plant architecture parameters. All data are expressed as mean ± standard deviation (SD).

## 3 Results

### 3.1 Effect of salinity and temperature on the growth of fungal isolates

Multifactorial ANOVA (three-factor model, Type III sums of squares) revealed that all three main factors — fungal isolate, temperature and NaCl concentration — had a highly significant effect on radial growth rate (p < 0.0001), as did all first- and second-order interactions. Their relative contributions, however, differed markedly. Isolate identity accounted for the largest single source of variation (SS = 272.489; 51.2% of the total sum of squares), reflecting the broad phenotypic diversity and physiological plasticity among the 28 isolates evaluated.

Among the two environmental factors, temperature exerted a considerably stronger main effect than salinity, accounting for 8.6% of the total variance (SS = 45.506; F = 2508.09; p < 0.0001) compared with 2.1% for NaCl concentration (SS = 11.367; F = 250.61; p < 0.0001) — a fourfold difference in explained variance — thereby identifying thermal regime as the dominant abiotic driver of fungal growth across the collection. The comparatively modest main effect of salinity is consistent with the ecological origin of these isolates, recovered from halophytic and xerophytic plants growing in saline, arid soils, and therefore expected to be halotolerant within the concentration range assayed.

Notably, the isolate × temperature interaction alone explained 27.2% of the total variance (SS = 144.715; F = 295.41; p < 0.0001), a greater proportion than either environmental factor individually, whereas the isolate × salinity and temperature × salinity interactions accounted for 5.1% and 0.3%, respectively. The significant three-way isolate × temperature × NaCl interaction (F = 8.39; p < 0.0001) indicates that salt tolerance is not a static physiological trait but is critically modulated by thermal conditions, suggesting a complex interplay between osmotic and thermal stress responses that varies in a genotype-specific manner. This hierarchy of effects provides the rationale for evaluating both stresses in combination rather than in isolation. Radial growth rates for each isolate under all temperature and salinity combinations are provided in **Supplementary Table 1**.

**Figure 1:**
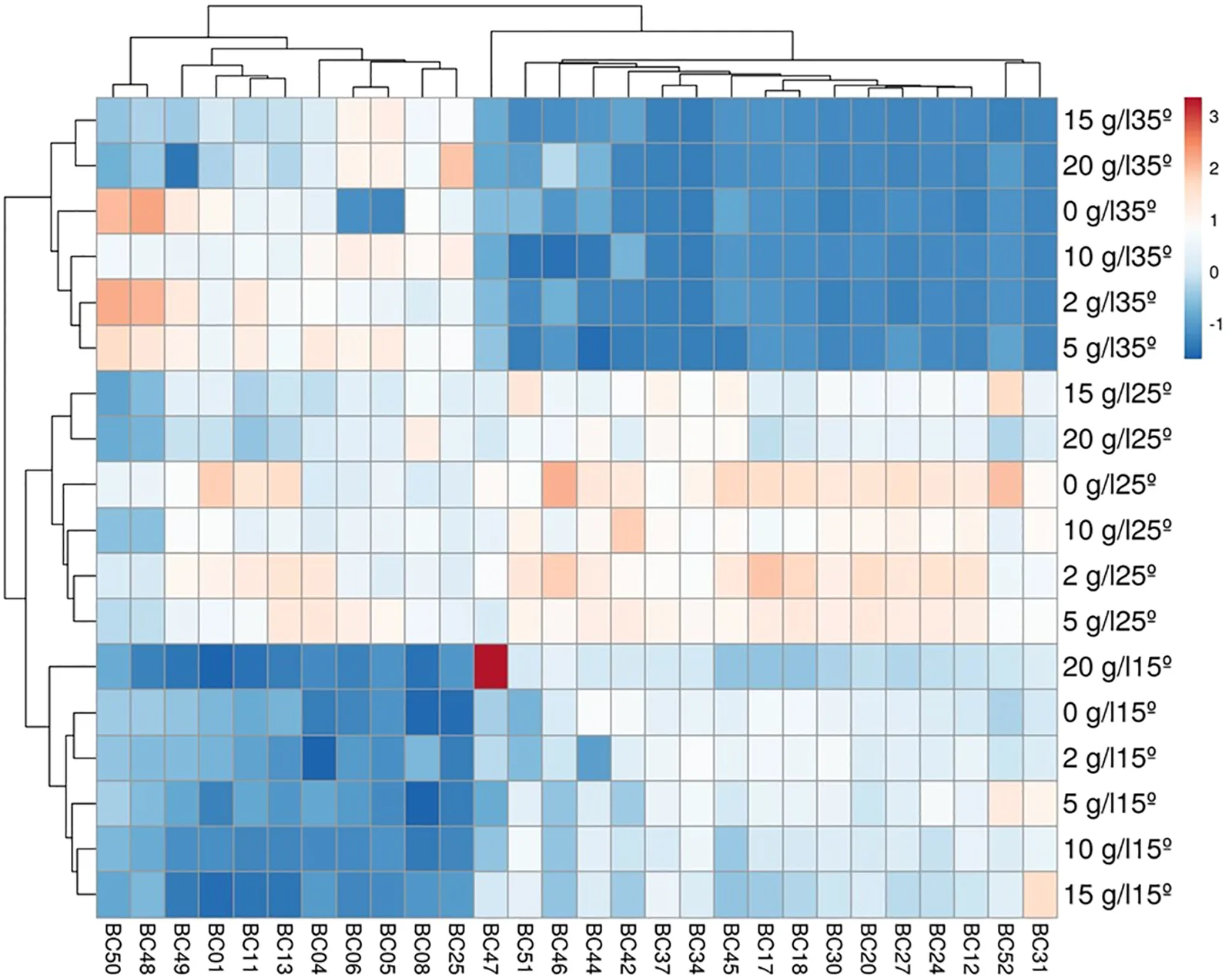
Hierarchical clustering heatmap of fungal isolate growth under combined thermal and saline stress. The matrix shows radial growth of 28 fungal isolates (columns) across 18 conditions (rows), combining three temperatures (15, 25, 35 °C) and six NaCl concentrations (0, 2, 5, 10, 15, 20 g L^–1^). Color intensity reflects Z-score normalized growth (red: above mean; blue: below mean). Rows and columns were clustered by Euclidean distance with average linkage. The row dendrogram highlights the dominant effect of temperature, with all 35 °C treatments clustering independently, while the column dendrogram groups isolates by their tolerance profiles across the combined stress gradient.

Hierarchical clustering analysis (**Figure 1**) grouped the 28 isolates into two clearly differentiated functional superclusters. Supercluster A comprised isolates with high physiological plasticity (BC50, BC48, BC49, BC01, BC11, BC13, BC04, BC06, BC05, BC08, and BC25), characterized by sustained growth under severe thermal stress (35 °C). Within this group, the BC05/BC06 pair (*Sordaria fimicola*) exhibited remarkable metabolic stability across the 15–25 °C temperature range. Supercluster B grouped the most stress-sensitive mesophilic isolates (BC51 to BC31), including the *Trichoderma* cluster (BC24, BC20, BC12), which showed near-total growth inhibition at 35 °C regardless of NaCl concentration. A notable phenotypic finding was the identification of isolate BC47 as a halotolerant specialist, displaying the highest positive deviation from the population mean (Z-score) under combined osmotic and low-temperature stress (15 °C and 20 g L^–1^ NaCl), suggesting a unique adaptation to osmotic stress at low temperatures. The row dendrogram further confirmed that temperature was the dominant selective factor, as all 35 °C treatments clustered independently of salinity regime.

**Figure 2:**
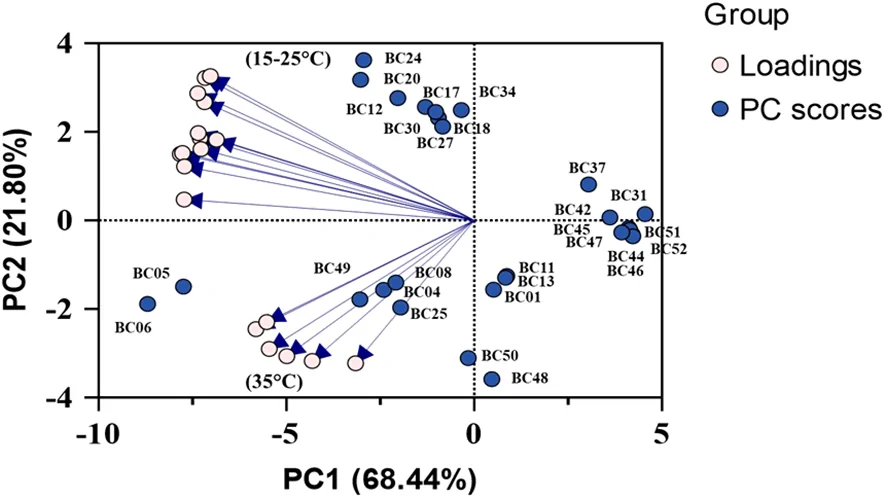
PCA biplot of fungal isolate growth performance under combined thermal and saline stress, accounting for 90.24% of total variance (PC1: 68.44%; PC2: 21.80%). Blue circles represent isolate scores; vectors indicate loadings of environmental variables (temperature and NaCl concentration). PC1 separates isolates by overall growth vigor, with high-performing genotypes (e.g., BC24, BC20, BC17) associated with moderate temperature vectors (15–25 °C). PC2 discriminates isolates by thermal stress response, with the close proximity of BC05 and BC06 (*Sordaria fimicola*) to the 35 °C vectors reflecting their nearly identical thermotolerant profiles.

Principal Component Analysis (PCA) accounted for 90.24% of the total variance (PC1: 68.44%; PC2: 21.80%) (**Figure 2**). PC1 discriminated isolates primarily based on overall growth vigor, with high-performing genotypes such as BC24, BC20, and BC17 positively associated with moderate temperature vectors (15–25 °C). PC2 separated a distinct subpopulation of stress-resilient isolates — BC05, BC06, BC48, and BC50 — oriented toward the 35 °C stress vectors. Isolates BC05 and BC06 (*Sordaria fimicola*) displayed nearly identical spatial coordinates, confirming their highly similar physiological profiles, consistent with the clustering pattern observed in **Figure 1**. Overall, the multivariate distribution revealed a clear divergence between the majority of isolates and a specific subset uniquely adapted to combined thermal and saline stress.

### 3.2 Effects of salinity and fungal inoculation on seed germination

**Figure 3:**
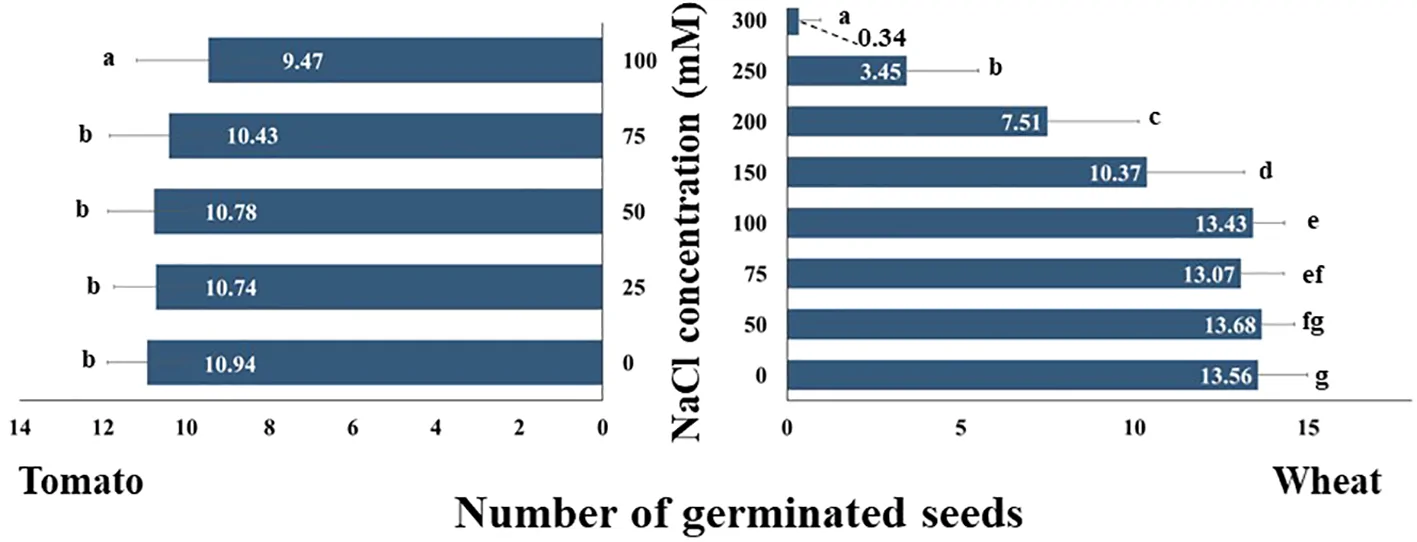
Effect of NaCl concentration on seed germination in tomato and wheat. Bars represent the mean number of germinated seeds (± SD) for tomato (*Solanum lycopersicum* L., left; *n* = 12) across a salinity gradient of 0–100 mM NaCl, and wheat (*Triticum aestivum* L., right; *n* = 16) across a salinity gradient of 0–300 mM NaCl. Different lowercase letters beside bars indicate statistically significant differences among salinity levels within each species (Tukey’s HSD test, *P* < 0.05).

**Figure 4:**
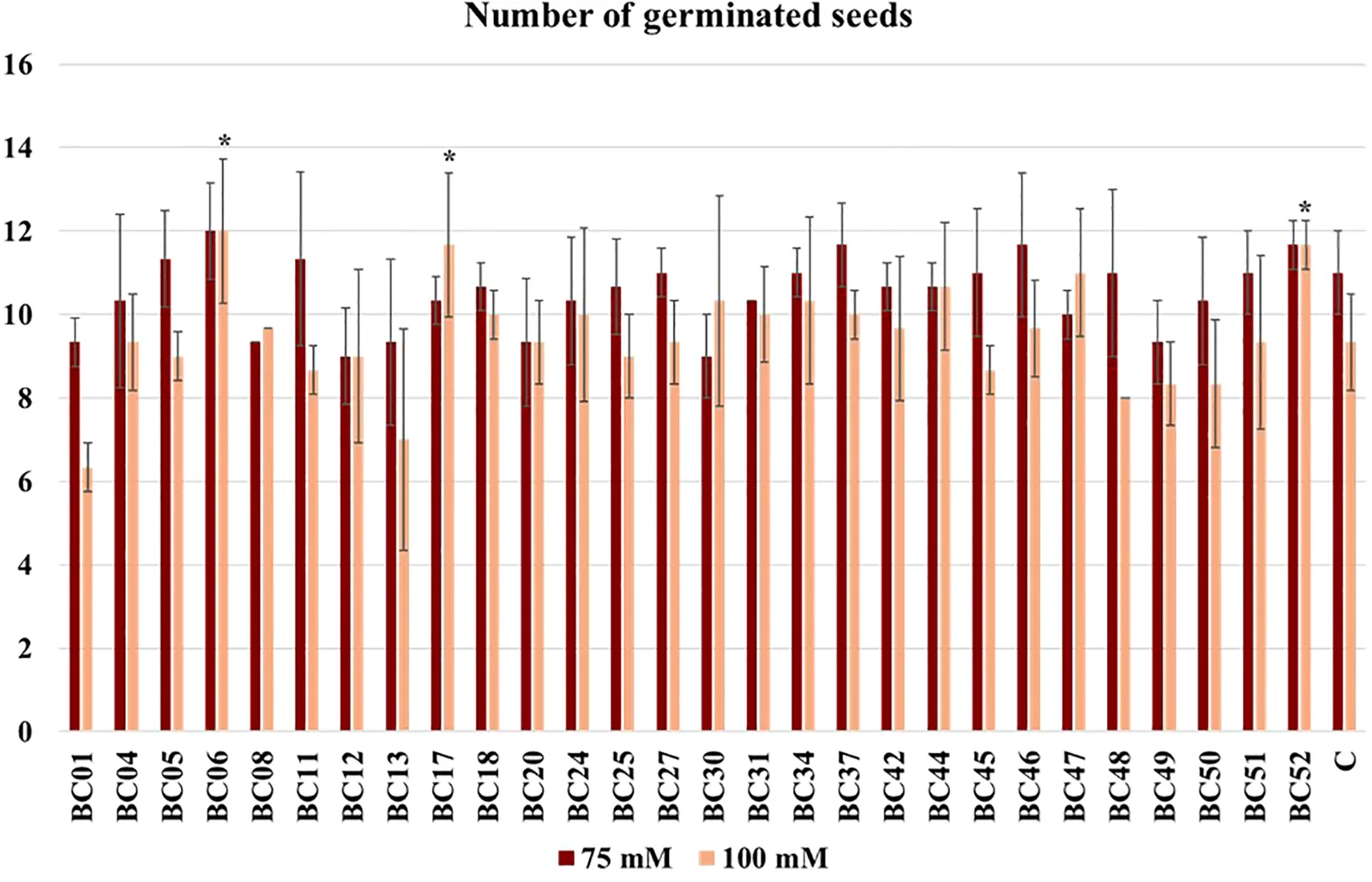
Bioprotective effect of endophytic fungal isolates on germinative capacity of *Solanum lycopersicum* L. under saline stress. Mean number of germinated seeds per treatment at 75 mM (dark red bars) and 100 mM (orange bars) NaCl, for isolates BC01–BC52 relative to the non-inoculated control (C). Error bars indicate SD (n = 3 × 12 seeds). Asterisks denote significant increases relative to the control within each NaCl concentration (Tukey’s HSD, *p* < 0.05).

Seed germination in both tomato (*Solanum lycopersicum* L.; *p* = 0.003) and wheat (*Triticum aestivum*; *p* = 0.0001) was significantly modulated by the interaction between fungal isolate and NaCl concentration (two-way ANOVA), indicating that bioprotective efficacy is strongly dependent on osmotic context (**Figure 3**). In tomato, the response model exhibited threshold-type behavior, in which low-to-moderate concentrations (0–75 mM NaCl) allowed the maintenance of physiological homeostasis and high germinative vigor, whereas 100 mM NaCl acted as a critical selective pressure, associated with statistically significant inhibition of radicle emergence. Consequently, this salinity level was selected as the optimal stress condition for discriminating the bioprotective potential of isolates in subsequent *in vivo* assays. Increasing osmotic pressure produced a clear differentiation in isolate efficacy, defining 75 mM NaCl as the inflection point for the selection of stable strains such as BC06 and BC52 (**Figure 4**; **Supplementary Table 2**), with no significant differences relative to the control. Under severe osmotic stress induced by 100 mM NaCl, the germinative capacity of the non-inoculated control underwent significant inhibition, evidencing the deleterious impact of salinity on seed water potential. Nevertheless, incipient symbiosis with specific fungal isolates exerted a robust mitigating effect, preserving germinative vigor through an active bioprotection mechanism. In this context, isolates BC06 and BC17 demonstrated superior biostimulant efficacy, maintaining radicle emergence rates virtually invariant across the saline gradient. Furthermore, the performance of BC52 and BC47 highlights their phenotypic plasticity and capacity to stabilize the seed microenvironment, effectively overcoming the salinity-induced limitations that compromised the remainder of the fungal collection.

**Figure 5:**
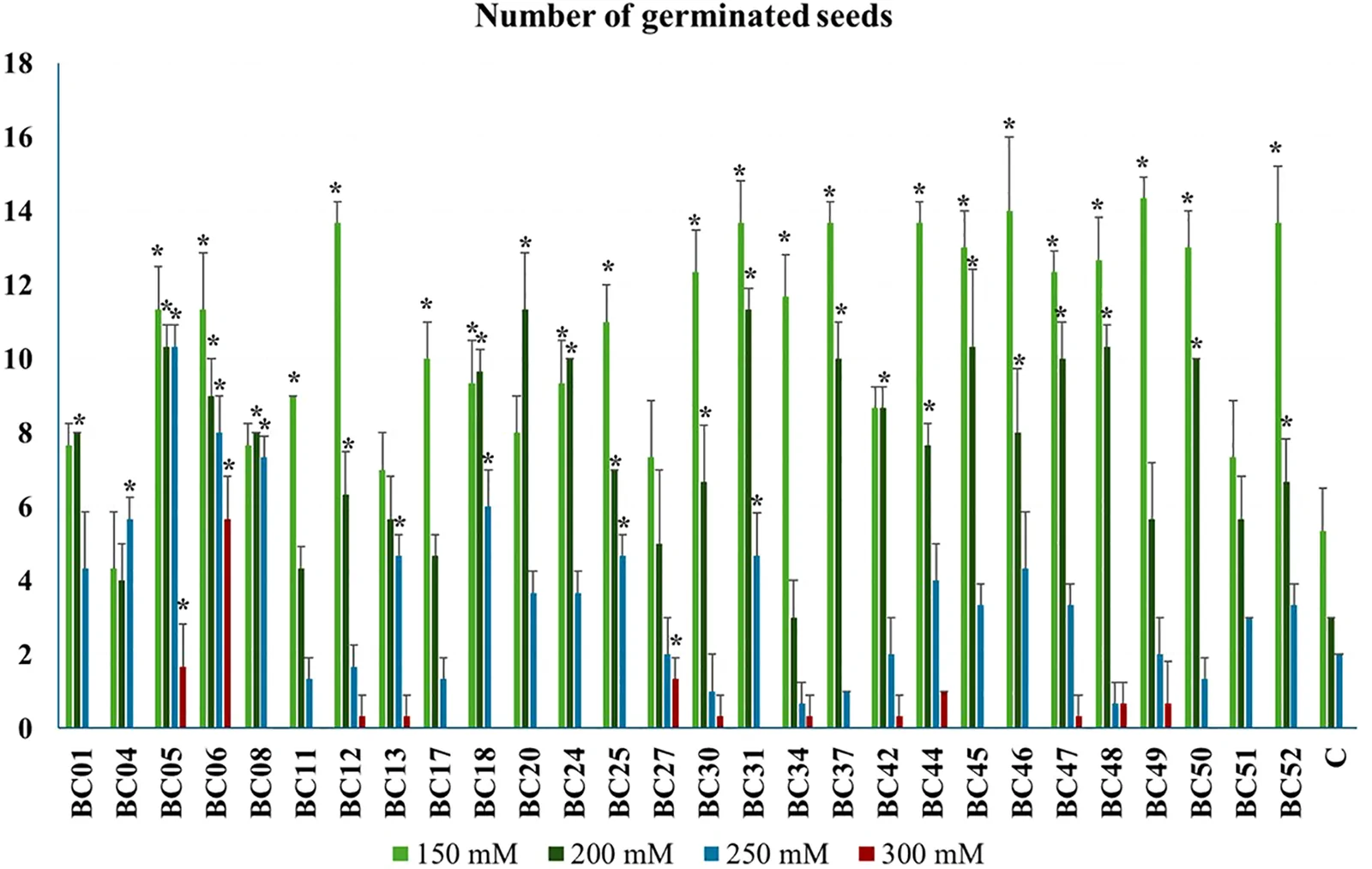
Bioprotective effect of endophytic fungal isolates on germinative capacity of *Triticum aestivum* under saline stress. Mean number of germinated seeds per treatment at 150 mM (dark blue), 200 mM (green), 250 mM (orange), and 300 mM (dark red) NaCl, for isolates BC01–BC52 relative to the non-inoculated control (C). Error bars indicate SD (n = 3 × 16 seeds). Asterisks denote significant increases relative to the control within each NaCl concentration (Tukey’s HSD, *p* < 0.05).

In wheat, inoculation with specific isolates not only mitigated the impact of NaCl, but significantly enhanced germinative capacity relative to the non-inoculated control (C) as stress severity increased. At the 150 mM inflection point (**Figure 3**), while the control experienced a marked reduction (5.33 ± 1.15), a substantial proportion of the fungal collection demonstrated a robust bioprotective effect, achieving germination levels significantly exceeding those of the control. Within this positive-response group, high-performing isolates BC49, BC46, and BC12 were particularly notable, increasing germinative capacity by 168.8%, 162.6%, and 156.4%, respectively, relative to the non-inoculated control (**Figure 5**; **Supplementary Table 3**). This promotion effect was even more pronounced under elevated stress conditions (200 mM NaCl). At this concentration, multiple isolates again broadly maintained seed viability, functioning as biological buffers against ionic toxicity. Among these, isolates BC20 and BC31 demonstrated exceptional rescue capacity, increasing germination success by 277.6% relative to the control. Other elite isolates, including BC05, BC45, and BC48, also sustained improvements exceeding 240%, consolidating their role as potent biostimulators of osmotic resilience across intermediate and high salinity ranges. Finally, at the 300 mM threshold, where control germination was completely abolished, isolate BC06 emerged as the sole treatment capable of overcoming the lethality barrier, maintaining a germinative capacity of 5.33 ± 1.15. This value not only represents a complete rescue from the 100% inhibition observed in the control but enables wheat under extreme stress to match the performance that the non-inoculated control achieves only at half the saline load (150 mM), substantially extending the tolerance limits of the species.

### 3.3 General impact of endophytic inoculation on seedling development under non-saline and saline conditions

To extend the evaluation beyond the germination stage, seedling development was assessed across the same experimental framework, examining the morphometric responses of both crop species to fungal inoculation under optimal (non-saline) and restrictive (saline) conditions (**Supplementary Figure 1**). Overall, inoculation elicited a wide spectrum of phenotypic responses, ranging from significant growth promotion and stress mitigation to growth trade-offs, depending on the specific isolate–host combination and salinity level. The following sections detail the morphometric changes and the statistical significance of these interactions for each crop species independently.

#### 3.3.1 Effect of fungal inoculation on tomato seedling development under non-saline and saline conditions

**Figure 6:**
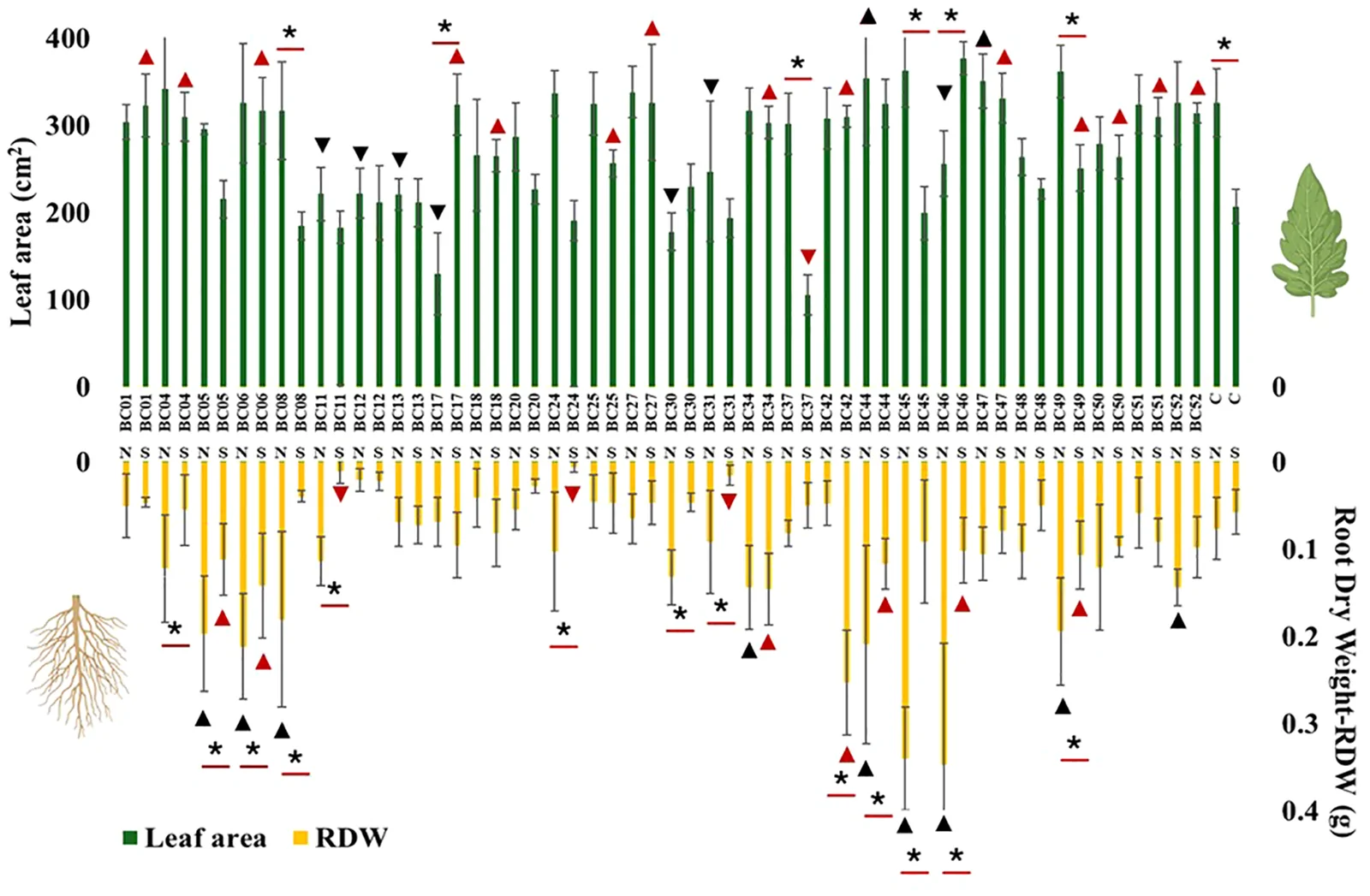
Effect of fungal inoculation on tomato seedling growth under non-saline and saline conditions. Leaf area (top) and root dry weight (bottom) of seedlings inoculated with 28 endophytic fungal isolates under non-saline (N) and saline (S) conditions. Bars show mean ± SD (n = 20). Two-way ANOVA revealed significant effects of isolate, salinity, and their interaction (*p* < 0.001); simple main effects were analyzed by fixing the salinity factor. Triangles indicate significance relative to the non-inoculated control (▲ higher, ▼ lower; black: non-saline, red: saline; Tukey’s HSD, *p* < 0.05). Asterisks with horizontal lines denote significant differences between N and S treatments for each isolate (*p* < 0.05). Absence of symbols indicates no significant difference.

The growth responses of tomato seedlings to fungal inoculation were strongly conditioned by the prevailing salinity level, as evidenced by the highly significant isolate × salinity interaction (I × S, p < 0.001) detected for both leaf area and root dry weight (RDW). Shoot dry weight, root dry weight and total biomass values for all isolates under both conditions are provided in **Supplementary Table 4**. While salinity severely compromised the growth of the non-inoculated control (C), several fungal isolates demonstrated a remarkable capacity to mitigate these deleterious effects. Regarding leaf area, most isolates did not exhibit a significant biostimulant effect under non-saline conditions (N), with the exception of BC44 (*Nothophoma anigozanthi*) and BC47 (*Nothophoma gossypiicola*), which significantly increased leaf area by 8.0% and 7.4%, respectively (**Figure 6**). Conversely, certain treatments resulted in a significant reduction in leaf area relative to the control, suggesting a potential metabolic cost of symbiosis in the absence of stress. This trend shifted markedly under saline stress (S), where the control underwent a pronounced decline in leaf area. Under these conditions, fourteen isolates significantly outperformed the saline control, with increases ranging from 21.34% (BC49, *Trichoderma saturnisporum)* to 60.0% (BC47, *N. gossypiicola*). Within this group, isolates BC01, BC06, BC17, BC27, and BC52 were particularly notable, achieving increases exceeding 50% and effectively maintaining a greater photosynthetic surface area despite salinity (**Supplementary Figure 2**). Notably, for several of these isolates, the absence of significant differences between their N and S treatments suggests a buffering mechanism that neutralized the impact of salt on leaf development.

**Figure 7:**
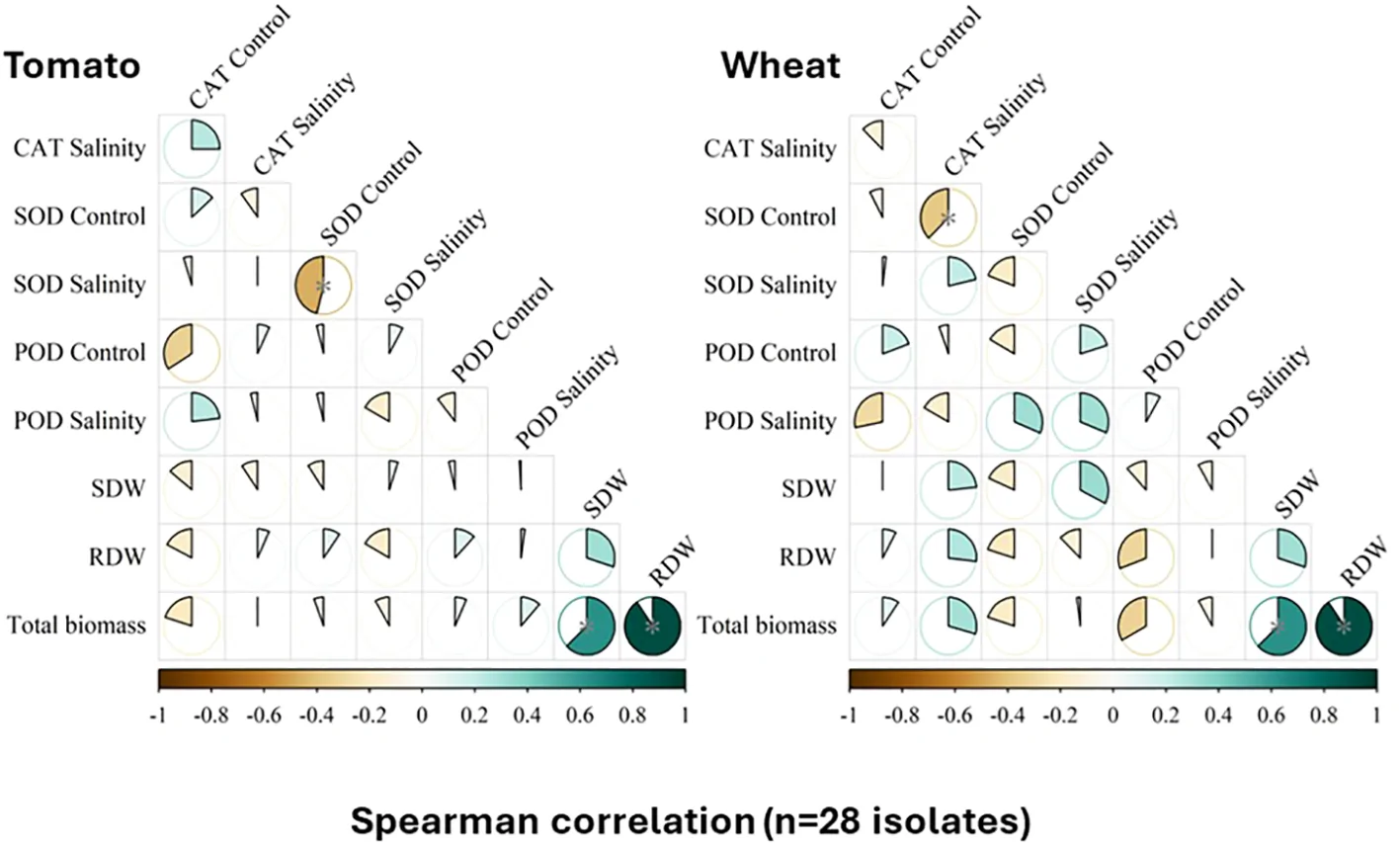
Spearman correlation analysis between antioxidant enzyme activities and plant biomass parameters in tomato and wheat. Correlation matrices display pairwise Spearman correlations between peroxidase (POD), catalase (CAT), and superoxide dismutase (SOD) activities under non-saline (Control) and saline (Salinity) conditions, and shoot dry weight (PSA), root dry weight (RDW), and total biomass across the 28 evaluated fungal isolates in tomato (left panel) and wheat (right panel). Pie chart size and color intensity represent the magnitude and direction of the correlation, with green indicating positive correlations and brown indicating negative correlations, as shown in the color scale (−1 to 1). Asterisks (*) denote statistically significant correlations (p < 0.05).

Shoot dry weight followed a more conservative pattern than leaf area. Under non-saline conditions, only BC45 (*N. gossypiicola*) and BC04 (*Macrophomina phaseolina*) significantly exceeded the control, increasing shoot dry matter by 107.8% and 86.8%, respectively (p < 0.05; **Supplementary Table 4**), whereas under saline stress no isolate differed significantly from the saline control. The biostimulant effect detected under salinity therefore operated primarily through the expansion of photosynthetic surface area and the preservation of root biomass rather than through an increase in shoot dry matter accumulation, a dissociation consistent with the leaf area responses described above and with the absence of a significant correlation between shoot biomass and antioxidant enzyme activity (**Figure 7**).

Regarding root development, RDW exhibited a differential response closely associated with isolate identity. Under non-saline conditions (N), most isolates maintained root biomass comparable to the control; however, a vigorous biostimulant effect was identified in isolates BC05 (159%), BC06 (176%), BC08 (135%), BC34 (88%), BC44 (173.2%), BC45 (343%), BC46 (352.3%), BC49 (153%), and BC52 (88%), suggesting an enhancement of basal root architecture. The impact of inoculation was more pronounced under saline stress (S), where the root system of the control underwent severe inhibition. Under these conditions, seven isolates acted as stress mitigators, preserving root biomass significantly superior to that of the control: BC05 (94%), BC06 (147%), BC34 (154%), BC42 (340.5%), BC44 (103%), BC46 (76.5%), and BC49 (86%). Notably, isolates that exhibited leaf area biostimulation, BC06 (*Sordaria fimicola*), BC34 (*Clarireedia narcissi*), BC42 (*Phoma* sp.), and BC49 (*T. saturnisporum*), also displayed consistent root biomass responses, increasing total biomass by 62.68%, 55.94%, 21.02%, and 31.09%, respectively, relative to the control under saline stress conditions (**Supplementary Table 4**).

**Figure 8:**
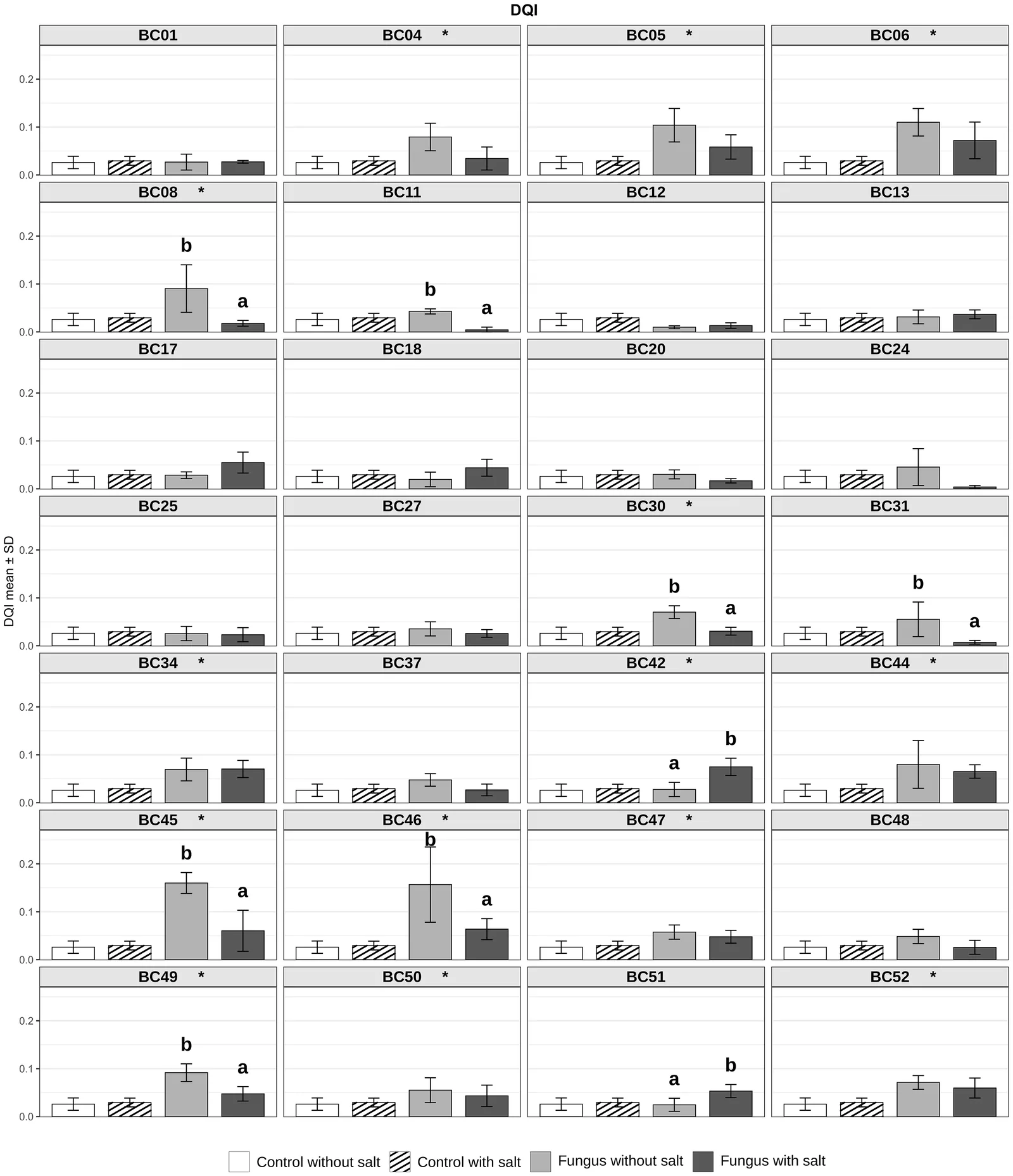
Dickson Quality Index (DQI) of tomato seedlings inoculated with endophytic fungal isolates under non-saline and saline conditions. Bars represent the mean ± SD (n = 20). Asterisks (*) above each isolate panel indicate overall significant differences relative to the non-inoculated control, as determined by multifactorial ANOVA (p < 0.05). Different lowercase letters above bars within the same isolate panel denote significant differences between non-saline and saline conditions (fungus without salt vs. fungus with salt), as determined by one-way ANOVA followed by Tukey’s HSD *post-hoc* test (p < 0.05). White and hatched bars represent non-inoculated controls under non-saline and saline conditions, respectively; light grey and dark grey bars represent fungus-inoculated treatments under non-saline and saline conditions, respectively.

To comprehensively assess seedling vigor and osmotic stress adaptability, the Dickson Quality Index (DQI) was calculated. This parameter, which integrates total biomass and the allometric relationships between root architecture and shoot, enables more precise discrimination of the survival potential of inoculated plants. Multifactorial analysis revealed that fungal inoculation significantly modulated plant quality (p < 0.001), with several isolates (indicated by asterisks in **Figure 8**) consistently outperforming the non-inoculated controls. Under non-saline conditions (N), the most prominent biostimulant effects were observed in seedlings inoculated with BC08 (*Macrophomina phaseolina*), BC11 (*Torula* sp.), BC30 (*Clarireedia narcissi*), BC31 (*Didymella longicolla*), BC45 (*N. gossypiicola*), BC46 (*N. gossypiicola*), and BC49 (*T. saturnisporum*), which attained the highest DQI values, suggesting that these fungi promote a highly efficient biomass distribution in the absence of stress. However, exposure to saline stress (S) significantly reduced their DQI, indicating that their biostimulant capacity is partially attenuated under ionic stress, although values remained superior to the non-inoculated control. In contrast, isolates BC42 and BC51 (*Phoma* sp.) displayed an inverse pattern, exhibiting significantly higher DQI values under saline conditions than under non-saline conditions, suggesting a stress-inducible bioprotective mechanism that is specifically activated in response to ionic stress.

The remaining isolates showing significant differences relative to the control, BC04, BC05, BC06, BC34, BC44, BC47, BC50, and BC52, did not exhibit significant differences between N and S conditions, reflecting a stable and salinity-independent biostimulant capacity that consistently enhanced seedling structural quality regardless of osmotic context. Collectively, these results reveal three functionally distinct response profiles within the fungal collection: isolates that maximize seedling quality under optimal conditions, isolates whose bioprotective effect is specifically induced by saline stress, and isolates that confer stable quality enhancement regardless of osmotic context, a diversity of strategies with complementary agronomic value for salinity-affected environments.

#### 3.3.2 Effect of fungal inoculation on wheat seedling development under non-saline and saline conditions

Shoot dry weight (SDW) in wheat was not significantly affected by salinity alone (*p* = 0.1153); however, fungal isolate identity significantly affected shoot biomass, and a highly significant isolate × salinity interaction was detected (*p* < 0.001), indicating that the wheat response to fungal colonization was strongly conditioned by the prevailing osmotic environment.

**Figure 9:**
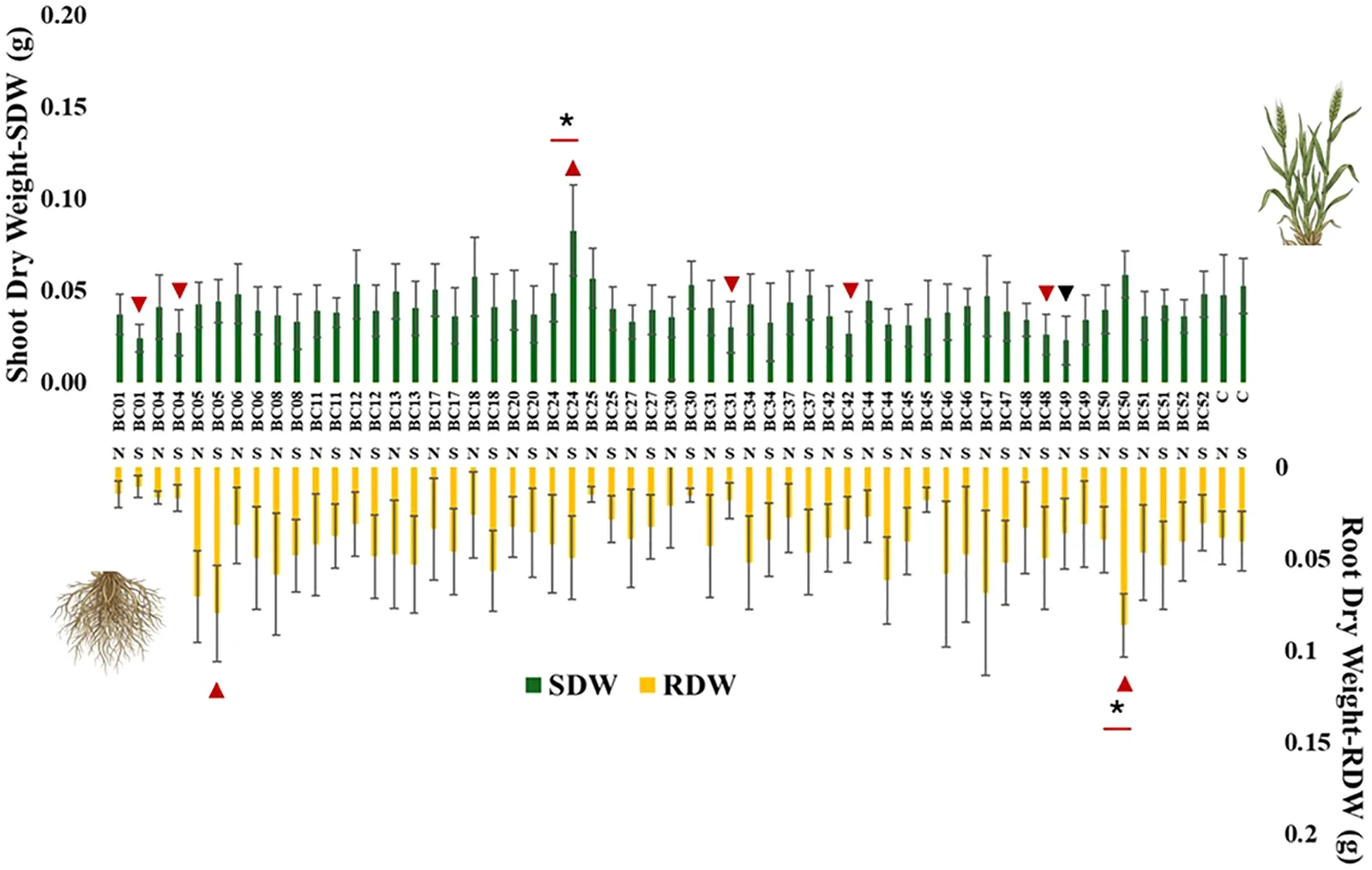
Effect of fungal inoculation on wheat seedling biomass under non-saline and saline conditions. Shoot dry weight (top, green bars) and root dry weight (bottom, yellow bars) of seedlings inoculated with 28 endophytic fungal isolates under non-saline (N) and saline (S) conditions. Bars show mean ± SD (n = 20). Symbols and statistical analysis as in **Figure 6**: triangles indicate significance relative to the non-inoculated control (▲ higher, ▼ lower; black: non-saline, red: saline; Tukey’s HSD, *p* < 0.05); asterisks with horizontal lines denote significant differences between N and S treatments (*p* < 0.05).

Under non-saline conditions (N), fungal inoculation in wheat elicited a predominantly neutral response in terms of shoot biomass. None of the 28 evaluated isolates achieved significant SDW promotion relative to the non-inoculated control (**Figure 9**). Conversely, BC49 induced a significant shoot biomass reduction exceeding 50%. Under saline stress (S), the wheat response to fungal inoculation was highly restrictive in terms of shoot biomass. Unifactorial analysis revealed that only isolate BC24 acted as an exceptional stress mitigator, increasing SDW by 58.1% relative to the saline control. The vast majority of isolates not only failed to compensate for the stress-induced growth reduction but also exhibited negative interactions with salinity, resulting in significant biomass losses. The most critical cases were BC01, BC04, BC31, BC48, and BC42, all of which showed reductions ranging from 44 to 52% relative to the non-inoculated saline control. These results suggest that wheat shoot biomass is highly sensitive to fungal colonization under ionic stress, where most isolates appear to exacerbate the metabolic cost of salinity, with the exception of specific genotypes such as BC24, which effectively preserved vegetative vigor (**Supplementary Figure 3**).

**Figure 10:**
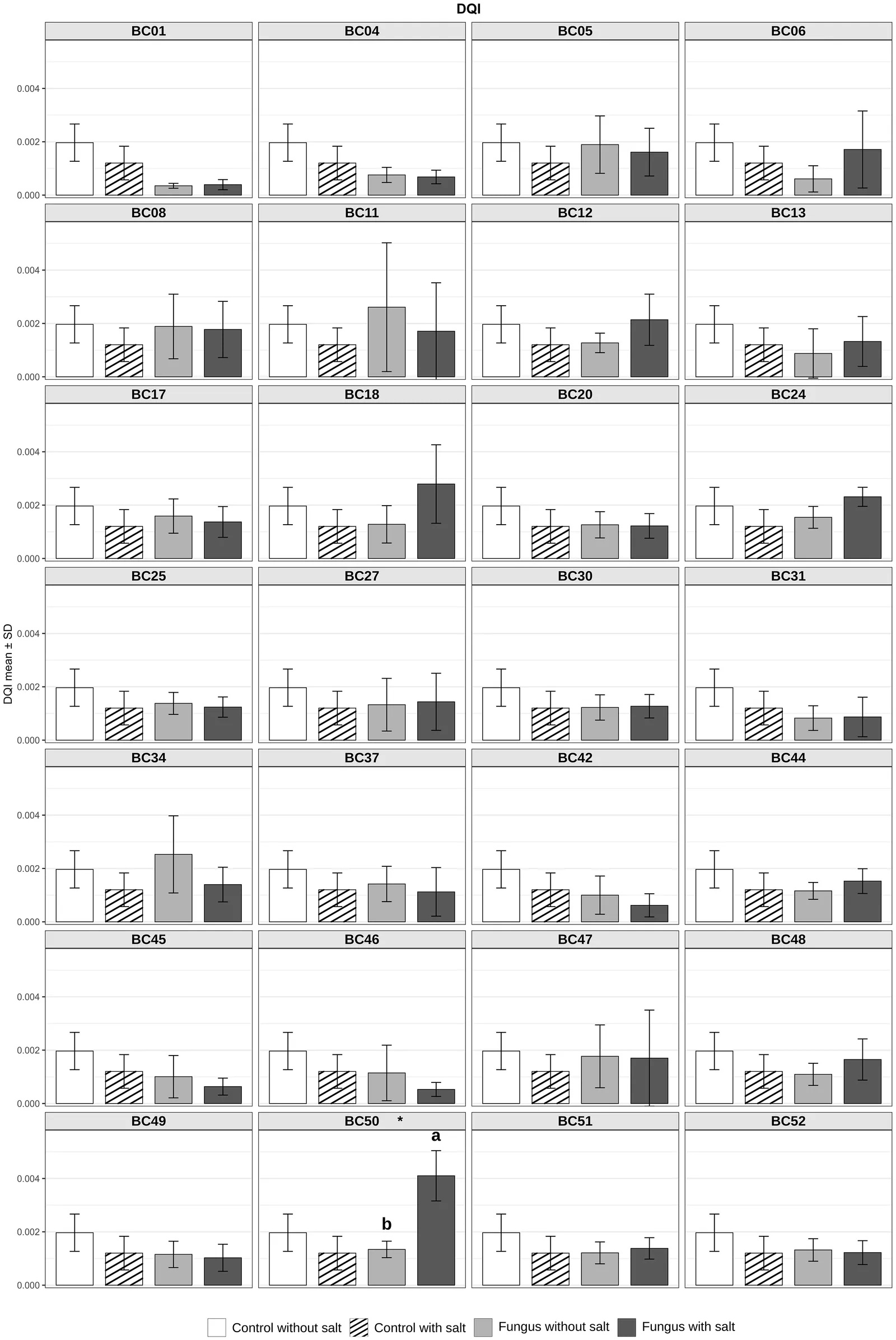
Dickson Quality Index (DQI) of wheat seedlings inoculated with endophytic fungal isolates under non-saline and saline conditions. Bars show mean ± SD (n = 20). Symbols and statistical analysis as in **Figure 8**: asterisks indicate significant differences relative to the non-inoculated control (multifactorial ANOVA, *p* < 0.05); different lowercase letters denote significant differences between non-saline and saline conditions within each isolate panel (one-way ANOVA, Tukey’s HSD, *p* < 0.05). White/hatched bars: non-inoculated control (non-saline/saline); light/dark grey bars: fungus-inoculated (non-saline/saline).

A broadly similar pattern was observed in the wheat root system. Multifactorial ANOVA revealed that both fungal isolate (p < 0.0001) and salinity level (p = 0.0453) exerted significant effects on root dry weight (RDW), and a highly significant interaction between both factors was detected (p < 0.0001), indicating that the influence of fungal inoculation on root growth varied differentially according to the saline stress applied. Nevertheless, RDW showed no significant variation across the majority of evaluated treatments, irrespective of salinity condition. Notable exceptions were isolates BC05 (*Sordaria fimicola*) and BC50 (*T. saturnisporum*), which induced a differential shift in root architecture under saline conditions, significantly increasing root biomass by 100% and 115%, respectively. While most fungal isolates maintained a neutral root response, these two isolates demonstrated a unique capacity to modulate RDW, clearly distinguishing themselves from the remainder of the evaluated collection. The Dickson Quality Index (DQI) showed limited significant differences among the evaluated treatments (**Figure 10**). Multiple comparison analysis using Tukey’s HSD test revealed that the vast majority of fungal isolates, under both non-saline and saline conditions, did not differ significantly from one another or from the non-inoculated controls, indicating that fungal inoculation had a generally neutral effect on seedling structural quality. The only notable exception was isolate BC50 (*T. saturnisporum*), which showed a significant difference between its non-saline and saline treatments with BC50S attaining the highest DQI value across the entire collection and differing significantly from most treatments including the saline control. This result suggests that BC50 harbors a stress-inducible bioprotective mechanism that specifically enhances seedling structural quality under ionic stress conditions. Pots experiments revealed contrasting responses between species. In tomato, fungal inoculation under saline stress enhanced leaf area by up to 60% and root dry weight by up to 340%. In wheat, responses were predominantly neutral or negative, with BC24 (*T. gamsii* as the sole shoot biomass mitigator (+58.1%) and BC50 (*T. saturnisporum*) uniquely improving the Dickson Quality Index under salinity.

This study provides the first evidence of *S. fimicola, C. narcissi*, and *N. gossypiicola* as effective biostimulants under saline stress, providing a robust framework for the rational selection of sustainable bioinoculants for salinity-affected agricultural systems.

Re-isolation of the inoculated strains from surface-sterilized roots confirmed their successful internal colonization in both crop species. Consistently, microscopic examination of ink-vinegar-stained root sections revealed the presence of fungal structures within the root cortex (**Supplementary Figure 4**), providing direct evidence of endophytic establishment and supporting the physiological responses observed under saline stress.

### 3.4 Antioxidant enzyme activity in response to fungal inoculation and saline stress

**Figure 11:**
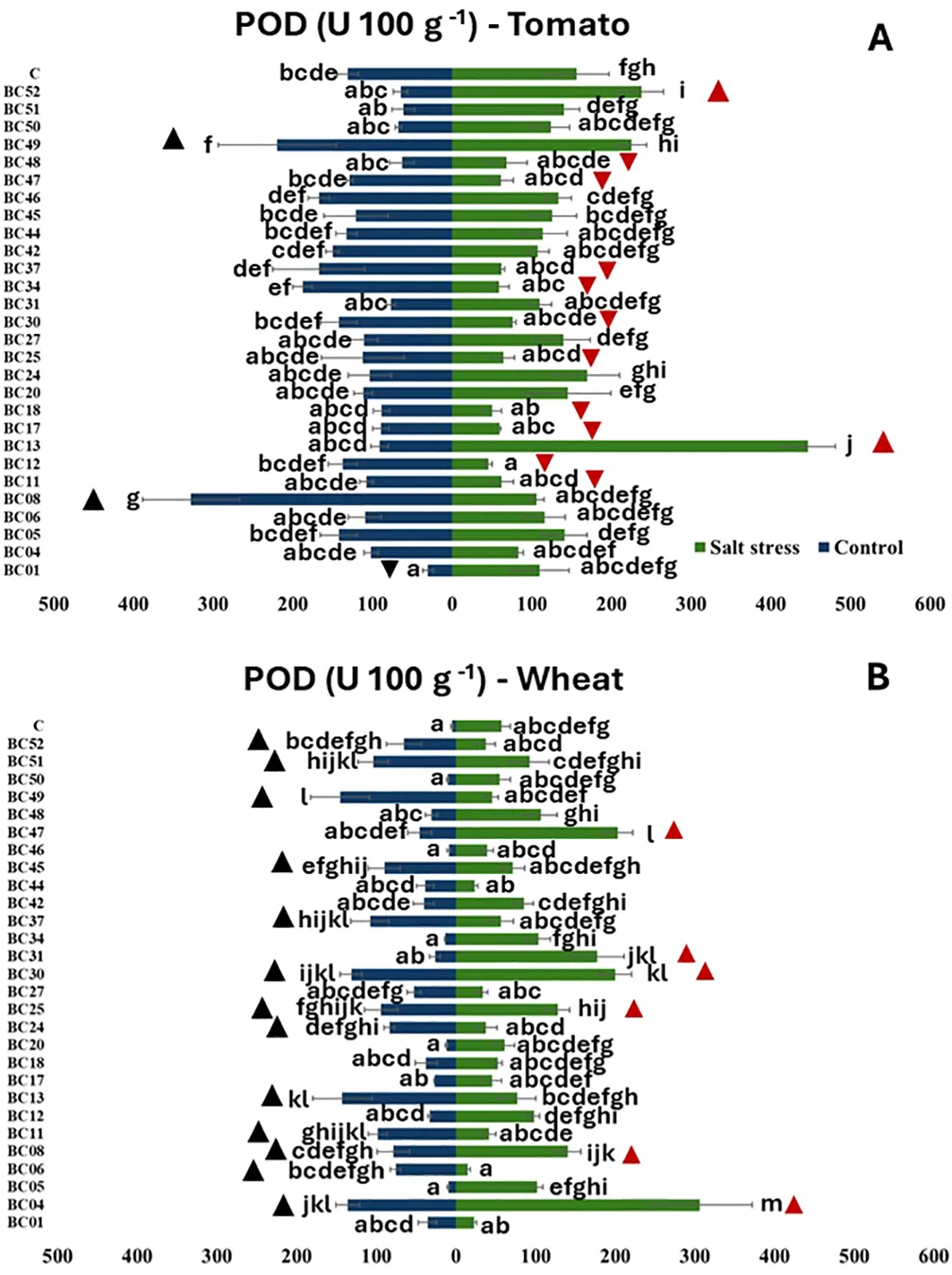
Effect of fungal inoculation on peroxidase (POD) activity in tomato and wheat seedlings under non-saline and saline conditions. POD activity (U·100 g^–1^ DW) in tomato **(A)** and wheat **(B)** seedlings inoculated with 28 endophytic fungal isolates under non-saline (control, blue bars) and saline (salt stress, green bars) conditions. Bars show mean ± SD (n = 3). Triangles indicate significance relative to the non-inoculated control (▲ higher, ▼ lower; black: non-saline, red: saline; Tukey’s HSD, *p* < 0.05). Lowercase letters denote homogeneous groups within each salinity condition.

**Figure 12:**
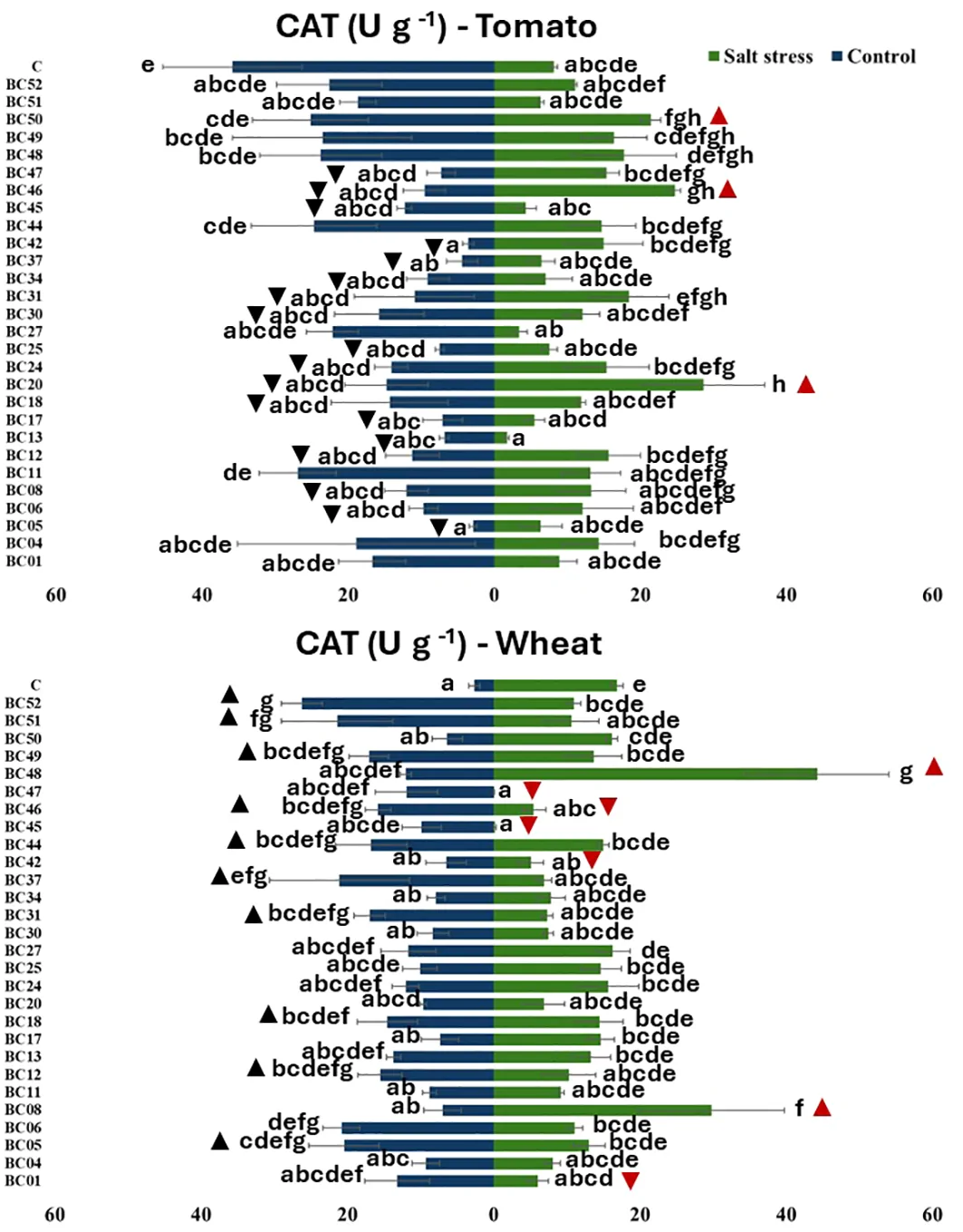
Effect of fungal inoculation on catalase (CAT) activity in tomato **(A)** and wheat **(B)** seedlings under non-saline (blue bars) and saline (green bars) conditions. CAT activity expressed as U g^–1^ DW. Bars show mean ± SD (n = 3). Symbols as in **Figure 11**: triangles indicate significance relative to the non-inoculated control (▲ higher, ▼ lower; black: non-saline, red: saline; Tukey’s HSD, *p* < 0.05); lowercase letters denote homogeneous groups within each salinity condition.

**Figure 13:**
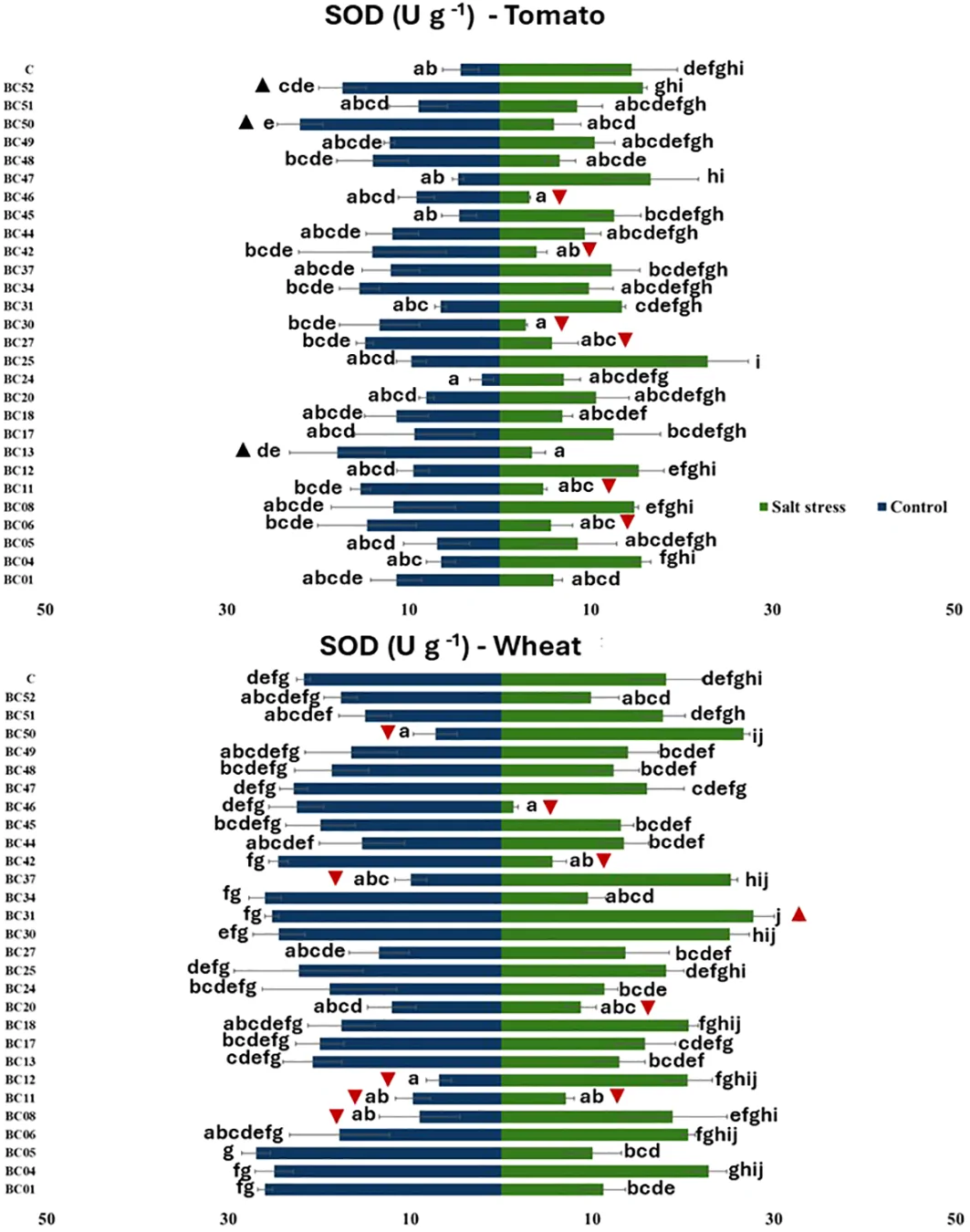
Effect of fungal inoculation on superoxide dismutase (SOD) activity in tomato **(A)** and wheat **(B)** seedlings under non-saline (blue bars) and saline (green bars) conditions. SOD activity expressed as U g^–1^ DW. Bars show mean ± SD (n = 3). Symbols as in **Figure 11**: triangles indicate significance relative to the non-inoculated control (▲ higher, ▼ lower; black: non-saline, red: saline; Tukey’s HSD, *p* < 0.05); lowercase letters denote homogeneous groups within each salinity condition.

The activity of the three antioxidant enzymes evaluated, peroxidase (POD), catalase (CAT), and superoxide dismutase (SOD), exhibited differentiated response patterns depending on the fungal isolate, crop species, and salinity conditions applied (**Figures 11**–**13**). Multifactorial ANOVA revealed significant effects of fungal isolate identity, salinity, and their interaction (I × S, p < 0.001) on the activity of all three enzymes in both crop species, indicating that the modulation of the antioxidant response was strongly dependent on the specific isolate–host combination and the prevailing osmotic context. Overall, the majority of isolates did not induce statistically significant differences relative to the non-inoculated control for any of the evaluated enzymes, suggesting that fungal colonization does not broadly disrupt the basal redox equilibrium of seedlings. However, specific isolate–enzyme–salinity combinations produced marked and statistically significant responses, revealing a highly context-dependent modulation of the antioxidant defense system within the evaluated collection.

In tomato, fungal inoculation had a limited overall effect on POD activity under non-saline conditions, with only BC08 and BC49 showing significant enhancement and BC01 a significant reduction relative to the non-inoculated control (**Figure 11A**). Under saline stress, the response became more polarized: while BC52 and BC13 significantly upregulated POD activity above the saline control, a considerable proportion of the collection showed significant suppression of this pathway, suggesting that ionic stress disrupts the peroxidase-mediated defense in most fungal symbioses evaluated. In wheat, the overall pattern was markedly different, with a broader and more consistent activation of POD across the collection under both conditions (**Figure 11B**). Under non-saline conditions, over half of the evaluated isolates significantly exceeded the control, indicating a constitutive stimulation of peroxidase activity by fungal colonization in this species. Under saline stress, this activating effect was maintained in a subset of isolates, with BC04 achieving the highest POD value across the entire collection — notably also one of the isolates showing enhancement under non-saline conditions — suggesting a stable and stress-reinforced biostimulant capacity for this antioxidant pathway.

In tomato, CAT activity showed a striking and generalized reduction under non-saline conditions, with 18 of the 28 evaluated isolates significantly suppressing enzyme activity relative to the non-inoculated control (**Figure 12A**). This widespread downregulation suggests that fungal colonization broadly modulates H_2_O_2_ metabolism under optimal growth conditions, possibly reflecting a rebalancing of the antioxidant machinery in the absence of oxidative stress. Under saline stress, this pattern was partially reversed, with isolates BC20, BC46, and BC50 significantly exceeding the saline control, indicating a selective upregulation of catalase-mediated H_2_O_2_ detoxification capacity specifically in response to ionic stress.

In wheat, the response was markedly different, with approximately 10 isolates significantly enhancing CAT activity relative to the non-inoculated control under non-saline conditions, suggesting a constitutive stimulation of this antioxidant pathway by fungal colonization in this species (**Figure 12B**). Under saline stress, the pattern shifted toward suppression in a subset of isolates, with BC01, BC42, BC45, BC46, and BC47 showing significantly reduced CAT activity relative to the saline control. In contrast, isolates BC08 and BC48 significantly exceeded the saline control, with BC48 attaining the highest CAT value across the entire wheat collection, representing the most potent activator of catalase-mediated defense under ionic stress.

In tomato, SOD activity showed a selective response to fungal inoculation under non-saline conditions, with isolates BC13, BC50, and BC52 significantly enhancing enzyme activity relative to the non-inoculated control (**Figure 13A**). Under saline stress, however, the pattern shifted toward suppression, with isolates BC06, BC11, BC27, BC30, BC37, and BC46 showing significantly reduced SOD activity relative to the saline control. This transition from activation under optimal conditions to suppression under ionic stress suggests that saline stress disrupts the superoxide-scavenging capacity mediated by these specific fungal symbioses, possibly reflecting a reorientation of the antioxidant response toward other ROS detoxification pathways.

In wheat, the overall SOD response was characterized by a generalized suppression, with all isolates showing significant differences relative to the control exhibiting reduced enzyme activity under both non-saline and saline conditions (**Figure 13B**). This consistent downregulation across conditions suggests that fungal colonization broadly attenuates superoxide dismutase activity in wheat, irrespective of osmotic context. The sole exception was isolate BC31, which significantly exceeded the saline control under ionic stress conditions, representing the only isolate capable of actively enhancing SOD-mediated superoxide scavenging in wheat under salinity.

## 4 Discussion

Among the major abiotic constraints threatening global food security, soil salinity stands out for its widespread impact on crop productivity, impairing germination, seedling establishment, and vegetative growth in strategically important species such as tomato and wheat through a combination of ionic toxicity, osmotic imbalance, and oxidative stress mediated by reactive oxygen species (ROS) (Mahmoud et al., 2025; Ndiate et al., 2022). Microbial biostimulants have emerged as a promising and sustainable approach to counteract these detrimental effects, enhancing germination success, early plant vigor, and overall performance by modulating key physiological processes including metabolism, water relations, and antioxidant defense (Gedeon et al., 2022; Mahmoud et al., 2025; García-Latorre and Poblaciones, 2024; Djebaili et al., 2020). The findings of the present study support this framework, suggesting that inoculation with endophytic fungi sourced from extreme coastal environments may help to alleviate saline stress-induced damage in both crop species, in line with a growing body of evidence showing that halotolerant endophytes isolated from halophytes or hypersaline habitats can significantly enhance germination, biomass accumulation, and yield under high NaCl concentrations or even seawater irrigation (Gupta et al., 2021; Nurrahma et al., 2024; Moghaddam et al., 2022; Airin et al., 2023; Aizaz et al., 2023; Badawy et al., 2021; Mutungi et al., 2024). However, the degree of protection conferred by endophytic inoculation was not uniform across isolates or salinity levels, but was strongly modulated by the physiological plasticity of each fungal genotype and the intensity of the osmotic challenge, consistent with meta-analytical evidence and experimental data reporting highly variable and context-dependent effects among endophytic isolates across osmotic gradients (Zhang et al., 2025; Moghaddam et al., 2022; Aizaz et al., 2023; Ma et al., 2025; Mutungi et al., 2024).

*In vitro* growth responses across the evaluated collection emerged from a multifactorial interaction between NaCl concentration and temperature rather than salinity alone, underscoring the complexity of stress tolerance in endophytic fungi and aligning with reports showing that halotolerant endophytes maintain stable growth only within relatively narrow thermal windows, with moderate temperature increases acting as a selective filter that determines which genotypes preserve metabolic stability under extreme osmotic conditions (Haseena et al., 2024; Dastogeer et al., 2018; Sangamesh et al., 2018; Ripa et al., 2019). Within this framework, two functionally contrasting adaptive strategies emerged that reflect the ecological specialization of the coastal microbiota. *Sordaria fimicola* (isolates BC05 and BC06, Supercluster A) exhibited a “thermal generalist” profile, sustaining stable growth rates and high metabolic robustness across the 15–25 °C range with minimal sensitivity to NaCl concentration, consistent with the broad salinity tolerance documented for Sordariomycetes in coastal marshes (Zhang et al., 2025) and for halophilic fungi optimizing their physiology within intermediate salinity ranges (Pérez-Llano et al., 2020; Ikiz et al., 2025). This homeostatic stability may underpin the subsequent efficacy of these isolates as germinative rescue agents under extreme saline stress. In contrast, isolate BC47 (*N. gossypiicola*) displayed a halotolerant specialist profile, attaining the highest Z-score values specifically under synergistic low temperature and high salinity stress, analogous to the extreme halotolerance reported in filamentous fungi from saline soils and hypersaline sediments (Ikiz et al., 2025; Yovchevska et al., 2025). *Trichoderma* isolates, however, showed pronounced vulnerability to thermal increases and saline conditions, consistent with the narrow mesophilic growth optima and dual sensitivity described for this genus (Cavalcante et al., 2025; Matrood and Rhouma, 2025; Andrés et al., 2022; Akbari et al., 2024). Collectively, the specific resilience of BC47 and the thermal stability of *S. fimicola* reinforce that salinity stress tolerance is not a uniform trait but a multidimensional adaptive capacity shaped by differentiated metabolic pathways, osmotic specialization versus thermal plasticity — as described for extremotolerant and extremophilic fungi (Ikiz et al., 2025; Pérez-Llano et al., 2020; Akbari et al., 2024; Zenteno-Alegría et al., 2024), with critical implications for the screening of climatically resilient next-generation biostimulants (Nurrahma et al., 2024; Ullah et al., 2021; Ali et al., 2022; Ikiz et al., 2024).

The germination patterns observed under NaCl stress in both crop species integrate coherently with the physiological behavior previously characterized during *in vitro* fungal growth assays, reinforcing the biological consistency of the screening pipeline applied in this study. The germination responses recorded in tomato and wheat confirm that seed germination success under saline conditions is strongly conditioned by the genotype × salinity × endophyte interaction, in line with biopriming studies involving *Trichoderma* and other fungal plant growth-promoting microorganisms (Cheng et al., 2023). In tomato, the threshold-type response pattern and the near-complete germination inhibition observed at 100 mM NaCl in the non-inoculated control are consistent with the complete suppression of germination reported at ≥75–100 mM NaCl in non-inoculated seeds in comparable studies (Mutungi et al., 2024). The capacity of isolates such as BC06 (*S. fimicola*), BC17 (*Clarireedia* sp.), BC52, and BC47 (*N. gossypiicola*) to preserve radicle emergence under this critical salinity threshold aligns with the germination and biomass enhancement reported following inoculation with endophytes belonging to *Penicillium, Ampelomyces*, and *Neocamarosporium* under saline conditions (Morsy et al., 2020; Moghaddam et al., 2021; Azad and Kaminskyj, 2016; Gaber et al., 2023). This contrasts with studies in which specific isolates of *T. asperellum* or *T. viride* clearly enhanced tomato germination under 50–100 mM NaCl or under non-saline conditions (Hu et al., 2025; Konappa et al., 2023; Iqbal et al., 2024; Mandal et al., 2023), suggesting that the biostimulant effect of *Trichoderma* on germination is highly strain- and context-dependent rather than a genus-level trait. It should be noted, however, that positive effects of fungal inoculation on germination are not universal: in several systems, inoculation benefits are primarily expressed as improvements in seedling vigor and post-germination survival without necessarily translating into a significant increase in the number of germinated seeds (De Souza et al., 2021; Lustosa et al., 2020; Khomari et al., 2018), a pattern fully consistent with the results obtained in the present study. In wheat, the marked “rescue effect” demonstrated by multiple isolates at 150–200 mM NaCl, and most remarkably, the ability of BC06 (*Sordaria fimicola*) to overcome the lethality barrier at 300 mM, reinforces the notion that endophytic fungi can not only mitigate but may also, in some cases, reverse the negative germination trajectory induced by saline stress, as has been described for halophilic fungal collections and individual strains such as *Penicillium chrysogenum* (Aizaz et al., 2023; Dargiri et al., 2025; Zhang et al., 2016). The strong genotype × salinity interaction observed across the collection is consistent with the intra-collection variability documented in other endophytic systems (Aizaz et al., 2023; Mutungi et al., 2024; Bouzouina et al., 2020), and suggests that the targeted selection of consortia adapted to specific osmotic windows represents a promising strategy for the design of germination biostimulants tailored to increasing salinization scenarios.

Among the isolates demonstrating effective buffering of saline stress in wheat, *Trichoderma gamsii* and *T. saturnisporum* deserve particular mention. *Trichoderma* species have consolidated as effective biological tools for mitigating saline stress across a range of crops, enhancing both early-stage performance — including germination and seedling establishment — and vegetative growth and key physiological parameters under NaCl conditions (Konappa et al., 2023; Cheng et al., 2023; Hu et al., 2025; Santos et al., 2025). The bioprotective capacity demonstrated here by these isolates thus extends the growing body of evidence supporting genus-level saline stress mitigation in *Trichoderma*, while also highlighting the intraspecific variability that conditions the magnitude of this effect across strains and osmotic contexts.

Taken together, these results confirm that coastal endophytes function as highly context-dependent biopriming agents capable of substantially shifting the salinity tolerance thresholds of sensitive crops, consistent with findings reported for halophilic fungi and endophytes isolated from saline habitats (Gupta et al., 2021; Bouzouina et al., 2020; Aizaz et al., 2023; Mutungi et al., 2024). The exceptional performance of BC06 across both tomato and wheat under extreme saline conditions positions *S. fimicola* as a priority candidate for the development of broad-spectrum biostimulant formulations targeting salinity-affected agricultural systems under the controlled conditions tested here, a potential that will require confirmation in soil and field environments.

Under saline conditions, beneficial fungi, particularly *Trichoderma* spp., endophytes, and arbuscular mycorrhizal fungi (AMF), have demonstrated the capacity to maintain crop growth and nutritional status through a combination of improved ionic homeostasis (↓Na⁺, ↑K⁺), enhanced photosynthetic efficiency, reinforcement of antioxidant enzyme systems (SOD, CAT, APX, POD), and accumulation of compatible osmolytes such as proline and soluble sugars, while simultaneously reducing ROS, MDA, and membrane damage (Porcel et al., 2011; Zhang et al., 2016; Ikram et al., 2019; Evelin et al., 2019; Diagne et al., 2020; Dar et al., 2022; Sabzi-Nojadeh et al., 2024; Bouskout et al., 2024; Tan et al., 2024; Pooja et al., 2024; Geng et al., 2025; Boorboori and Lackóová, 2025; Hu and Bidochka, 2025; Lalthazuali et al., 2025). Nevertheless, these effects are consistently reported as highly dependent on fungal strain identity, host species, and salinity level, with neutral, negative, or strongly positive responses arising from the specific fungus–plant–stress intensity interaction rather than representing a universal biostimulant effect (Evelin et al., 2019; Ikram et al., 2019; Sánchez-Montesinos et al., 2019; Wahab et al., 2023; Hu and Bidochka, 2025; Boorboori and Lackóová, 2025). The results of the present study fit fully within this framework, revealing broad functional diversity within the evaluated collection and markedly differentiated response patterns between tomato and wheat. t should be emphasized that these mechanisms (ionic homeostasis, osmolyte accumulation, membrane protection, antioxidant reinforcement) derive from the cited literature and were not directly measured in the present study; they are therefore discussed here only as plausible explanatory hypotheses.

In tomato, salinity emerged as a pivotal modulator of the plant response to fungal inoculation, reinforcing previous findings reported for endophytes associated with halophytic shrubs and the tomato rhizosphere (Kazerooni et al., 2022; Mutungi et al., 2024; Mekkaoui et al., 2024). Under non-saline conditions, most isolates exerted neutral or even slightly negative effects on leaf area, suggesting a metabolic cost associated with symbiosis establishment in the absence of environmental stress — a pattern previously documented for *Trichoderma* and AMF, which rarely improve biomass or the Dickson Quality Index (DQI) under optimal growth conditions (Mekkaoui et al., 2024; Leuratti et al., 2025). Under saline conditions, however, a substantial subset of isolates significantly enhanced both leaf area and root dry weight (RDW), effectively attenuating the growth penalty imposed by NaCl. The magnitude and direction of these responses are consistent with the biomass increases of 30–60% or more reported for endophytes such as *Actinomucor elegans* and *Podospora bulbillosa*, as well as for several AMF species in tomato exposed to 100–150 mM NaCl (Al-Karaki, 2006; Heidarianpour et al., 2020; Kazerooni et al., 2022; Mutungi et al., 2024; Mekkaoui et al., 2024). Particularly noteworthy is the subset of isolates designated here as “buffering” isolates — those showing no significant differences between saline and non-saline conditions — which demonstrated the capacity to maintain growth and photosynthetic efficiency across both osmotic contexts, a response typically associated with reductions in H_2_O_2_ and MDA levels and enhanced antioxidant activity (variables not quantified in the present study) (Al-Karaki, 2006; Gupta et al., 2021; Mekkaoui et al., 2024).

The marked stimulation of root biomass and DQI in tomato aligns with evidence that *Trichoderma* and other plant growth-promoting fungi (PGPF) remodel root architecture and improve seedling structural quality parameters, increasing root weight even in the absence of salinity (Leuratti et al., 2025; Khoshmanzar et al., 2019; Xiang et al., 2023). Isolates that optimized DQI under non-saline conditions but showed attenuated effects under stress resemble the behavior described for *T. asperellum* and AMF in tomato seedlings, where improvements in leaf number, stem diameter, and root length are not always reflected in proportional increases in total biomass (Leuratti et al., 2025; Khoshmanzar et al., 2019). In contrast, isolates BC42 and BC51, which exhibited superior DQI exclusively under saline conditions, are consistent with the profile of “stress-inducible” symbionts, as described by Gupta et al. (2021) where biomass and quality improvements occur primarily under NaCl and are associated with antioxidant activation, hormonal regulation, and maintenance of ionic homeostasis; in our case these specific mechanisms were not assessed and are proposed only as hypotheses. This functional classification into three profiles — optimal, stress-inducible, and stable across both osmotic contexts — coincides with the diversity of adaptive strategies proposed in the literature on endophytes and AMF under saline conditions (Mekkaoui et al., 2024; Gupta et al., 2021; Aizaz et al., 2023; Eftekhari et al., 2025).

The contrast with wheat results is particularly striking and adds important nuance to the existing literature. While numerous studies report consistent increases in height, biomass, water content, and photosynthetic pigments in wheat inoculated with *Trichoderma longibrachiatum* T6, *T. reesei*, and *Penicillium chrysogenum*, among others, under 100–150 mM NaCl (Zhang et al., 2016; Ikram et al., 2019; Bouzouina et al., 2020; Elgharably and Nafady, 2021; Dargiri et al., 2025), the majority of isolates evaluated in the present study exerted neutral or even detrimental effects on shoot biomass under saline stress, with BC24 emerging as the sole isolate acting as a clear stress mitigator. These predominantly neutral or negative responses may also reflect alternative, non-exclusive explanations, such as a metabolic cost of symbiosis establishment, isolate–cultivar incompatibility, or effects imposed by the substrate and experimental conditions, rather than an intrinsic lack of biostimulant capacity, underscoring the context-dependent complexity of plant–fungus interactions (Bouzouina et al., 2020; Elgharably and Nafady, 2021; Aizaz et al., 2023; Miranda et al., 2023).

The root biomass response in wheat — predominantly neutral across the collection, with the notable exceptions of BC05 (*S. fimicola*) and BC50 (*T. saturnisporum*), which doubled or exceeded root biomass under saline conditions, is consistent with studies in which only specific AMF or *Penicillium/Fusarium* strains significantly increased root dry weight and grain yield at 150 mM NaCl, simultaneously reducing Na⁺ and Cl⁻ tissue concentrations (Elgharably and Nafady, 2021; Aizaz et al., 2023). The fact that only BC50 (*T. saturnisporum)* showed a clear DQI increase under salinity reproduces the behavior observed in highly adapted consortia, such as AMF + *Penicillium funiculosum and Fusarium oxysporum* or *Penicillium* CM022, which markedly elevate biomass, pigment content, antioxidant activity, and yield under high NaCl, while most other treatments remain neutral (Elgharably and Nafady, 2021; Dargiri et al., 2025).

Collectively, these data position fungal-mediated salinity tolerance improvement as a consistent outcome in tomato but an exception in wheat, with the magnitude and direction of the response strongly conditioned by isolate identity, host species, and stress intensity — as highlighted by both experimental studies and recent reviews on endophytes and halophilic fungi (Gupta et al., 2021; Aizaz et al., 2023; Sabzi-Nojadeh et al., 2024; Kapadia et al., 2021). The identification of isolates with opposing effects in tomato and wheat further reinforces the concept of plant–microorganism compatibility specificity and supports the widely advocated need for precise selection of crop- and salinity gradient-adapted consortia prior to large-scale agronomic implementation (Gupta et al., 2021; Aizaz et al., 2023; Miranda et al., 2023; Kapadia et al., 2021).

Plant antioxidant defense under salt stress encompasses both enzymatic and non-enzymatic components, including ascorbate, glutathione, carotenoids, tocopherols, and flavonoids, which can interact with different ROS species or support enzymatic function (Ighodaro et al., 2018; Liu, 2010; Zandi and Schnug, 2022; Rudenko et al., 2023). The present study focuses specifically on antioxidant enzymes (POD, CAT, SOD), given their central role in the controlled degradation of ROS and their status as the analytical focus of this work (Huchzermeyer et al., 2022; Ighodaro et al., 2018; Ali et al., 2020). This scope delimitation does not imply that non-enzymatic components are functionally irrelevant, but rather reflects the analytical boundaries of the present dataset; their interpretation should therefore be considered within the broader context of redox homeostasis, in which enzymatic activity represents only one layer whose significance depends on the physiological context and its balance with other antioxidant systems (Lei et al., 2015; Michiels et al., 1994). The differential modulation of antioxidant enzyme activities further underscores the complexity of endophyte–host interactions under saline stress. Consistent with evidence that endophytic fungi fine-tune specific combinations of POD, CAT, and SOD rather than activating a uniform antioxidant program (Gupta et al., 2021; González‐Teuber et al., 2022; Nurrahma et al., 2024), the highly isolate- and species-specific patterns recorded here fit within the broader evidence of contrasting responses within the same crop. Isolates exhibiting a constitutive antioxidant profile maintained significantly elevated POD activity independently of the prevailing osmotic context, a response pattern that resembles fungal consortia sustaining antioxidant activity regardless of stress intensity and represents a particularly valuable trait for bioinoculant applications in environments subject to variable salinity regimes (González‐Teuber et al., 2022; Irshad et al., 2023; Zhu et al., 2025). The more selective, stress-dependent responses in tomato are consistent with studies in Solanaceae where specific endophytes strongly activate POD and SOD under NaCl while others barely modify the enzymatic response despite improving tolerance (Zou et al., 2023; Farahat et al., 2020; Berková et al., 2024). The widespread CAT suppression in tomato under non-saline conditions is best interpreted as a rebalancing of H_2_O_2_ homeostasis under low oxidative load rather than a detrimental colonization effect, with other H_2_O_2_ sinks compensating to maintain redox signaling at non-toxic levels (Willekens et al., 1997; Gupta et al., 2021; Nurrahma et al., 2024), while the selective CAT activation observed under saline stress aligns with a stress-inducible detoxification model (González‐Teuber et al., 2022; Sahu et al., 2021; Berková et al., 2024). The near-universal SOD attenuation in wheat — with a single isolate as the sole exception, reflects the strong isoenzyme-specific regulation of SOD families in cereals under ionic stress (Jiang et al., 2018), and cases where reduced enzymatic activities accompany improved growth likely reflect reduced ROS load from enhanced ionic homeostasis or redirection toward non-enzymatic antioxidant pathways rather than weakened defenses (Zou et al., 2023; Zhu et al., 2025; Jan et al., 2019; Nurrahma et al., 2024).

The enzyme data across the evaluated collection thus reveals three functionally distinct profiles. A first group exhibited a constitutive antioxidant profile, maintaining significantly elevated POD activity independently of the prevailing osmotic context. A second group showed a stress-inducible profile, with enzymatic activation occurring exclusively or predominantly under NaCl, suggesting that ionic stress acts as a specific trigger for the upregulation of distinct antioxidant pathways in these isolate–host combinations. A third group exhibited relative enzyme suppression under salinity, possibly reflecting a redirection of the antioxidant response toward non-enzymatic defense mechanisms or a reduction in ROS load derived from improved ionic homeostasis (Gupta et al., 2021; Sahu et al., 2021; Zou et al., 2023; Maslennikova et al., 2023; He et al., 2026). These profiles, integrated with the germination, growth, and root architecture data, provide a robust framework for the rational selection of elite isolates with complementary modes of action for sustainable bioinoculant development in salinity-affected agricultural systems (Gupta et al., 2021; Zhu et al., 2025; Nurrahma et al., 2024; Maslennikova et al., 2023).

The Spearman correlation analysis revealed that antioxidant enzyme activity is not a universal predictor of plant biomass across the evaluated collection, a finding consistent with the broader literature showing that the relationship between antioxidant enzymes and growth is neither linear nor constant (**Figure 7**). Enzyme activities such as SOD, CAT, and POD typically respond strongly to abiotic stress — including salinity — while growth parameters simultaneously decline, so that higher antioxidant activity may reflect greater stress tolerance or reduced oxidative damage rather than directly promoting biomass accumulation (Pour-Aboughadareh et al., 2020; Hou et al., 2021b; Gao et al., 2008; Abogadallah et al., 2009; Lei et al., 2025; Yan et al., 2018; Penella et al., 2016). Meta-analyses and multi-treatment studies further confirm that enzyme–growth correlations are highly dependent on stress type, species, and treatment, ranging from positive to weak or non-significant depending on context (Hou et al., 2021a; Lei et al., 2025; Alam et al., 2023; Li et al., 2024; Kidwai et al., 2020; Jebara et al., 2005). In this framework, the absence of significant enzyme–biomass correlations in both crops suggests that antioxidant enzymes functioned primarily as redox adjustment markers rather than direct growth determinants. The sole exception — the positive correlation between constitutive POD activity and root dry weight in tomato — is consistent with the well-known role of class III peroxidases in cell wall remodeling and root development (Mahmood et al., 2021; Yıldız and Aki, 2025) explaining its specific association with root biomass while other enzymes remained decoupled from this trait. The species-specific reorganization patterns detected — negative SOD correlation across salinity levels in tomato, and compensatory CAT–SOD and inter-condition CAT relationships in wheat — further underscore the host-specific nature of endophyte-mediated antioxidant modulation under saline stress (Zou et al., 2023; Nurrahma et al., 2024).

A number of limitations should be acknowledged when interpreting these results. First, the present study is essentially correlational: it documents robust associations between fungal inoculation and germination, growth, root architecture, and antioxidant enzyme activity, but does not provide direct experimental evidence for the underlying mechanisms. Consequently, the processes invoked throughout this discussion — improved ionic homeostasis, osmolyte accumulation, hormonal regulation, membrane protection, and non-enzymatic antioxidant pathways — should be regarded as plausible, literature-supported hypotheses rather than mechanisms demonstrated here, and alternative explanations cannot be excluded. Second, all assays were conducted under controlled conditions, with defined substrates, fixed salinity levels, and a limited number of genotypes; the performance of these isolates under field conditions — across different soil types, native microbial communities, fluctuating environmental regimes, and a wider range of crop cultivars — remains unknown. Future work should therefore combine targeted mechanistic assays (e.g., Na⁺/K⁺ ratios, ROS and lipid-peroxidation markers, osmolyte and phytohormone profiling, and gene-expression analyses) with multi-site, multi-season field trials before these isolates can be recommended for large-scale agronomic use.

Among the most relevant findings of the present study is the identification of poorly characterized fungal species, including *Sordaria fimicola*, *Clarireedia narcissi*, and *Nothophoma gossypiicola*, as effective biostimulants under saline stress conditions. To the best of our knowledge, no previous reports document these species as biostimulant endophytes in tomata or wheat, and the present work would therefore constitute the first record of their capacity to promote plant growth and attenuate salinity-induced damage.

Consistent with the literature describing endophytic fungi as modulators of ionic homeostasis, antioxidant systems, and gene expression under abiotic stress (Waqar et al., 2023; Gupta et al., 2021; Verma et al., 2022; Ma et al., 2025), *S. fimicola* — primarily known as a coprophilous saprotrophic fungus (Arif and Saleem, 2017; Cain and Groves, 1948), recently reported as a causal agent of disease in olive (Petrović et al., 2024) and as an antagonist of plant pathogens (Wiewióra et al., 2024) — demonstrated consistent performance across multiple experimental stages in the present study, from germinative rescue under extreme salinity to root biomass promotion and antioxidant modulation, positioning it as a functionally versatile endophyte of considerable biotechnological interest.

Similarly, *C. narcissi* and *N. gossypiicola*, described in the literature primarily in the context of plant disease, exhibited a clear biostimulant capacity under saline stress in the present study. This is consistent with the concept of a pathogen–mutualist continuum in root-associated fungi, whereby changes in host identity, nutritional status, or stress level can shift the outcome of the interaction from pathogenic to beneficial (Redkar et al., 2022; Hacquard et al., 2016; Ujimatsu et al., 2025). Collectively, these results reinforce the need to explore non-conventional fungal taxa — including those sourced from saline or hypersaline environments — as novel sources of halotolerant endophytic fungi with biostimulant potential (Ma et al., 2025; Aizaz et al., 2023).

## 5 Conclusion

This study demonstrates that endophytic fungi from saline coastal ecosystems harbor significant and functionally diverse biostimulant potential under saline stress. Salt tolerance emerged as a multidimensional adaptive capacity critically shaped by the interaction between osmotic and thermal stress, highlighting the value of multi-stress screening for climatically resilient bioinoculant identification. *Sordaria fimicola* (BC06) stood out as the most versatile isolate, preserving radicle emergence in tomato at 100 mM NaCl and uniquely overcoming complete germination inhibition in wheat at 300 mM NaCl. Greenhouse experiments revealed contrasting host-specific responses, with tomato showing broad growth promotion and wheat predominantly neutral or negative reactions, reinforcing the need for crop-adapted bioinoculant selection. Three functionally distinct antioxidant profiles — constitutive, stress-inducible, and enzyme-suppressive — reflected the diversity of ROS management strategies within the collection. Notably, this study provides the first evidence of *S. fimicola, C. narcissi*, and *N. gossypiicola* as effective biostimulants under saline stress, expanding the known repertoire of beneficial endophytic fungi and highlighting the biotechnological potential of non-conventional fungal taxa from extreme coastal environments for the development of sustainable bioinoculants in salinity-affected agricultural systems.

## Data Availability

The datasets generated and analysed during this study are included in the article and its **Supplementary Material**. The ITS sequences of the fungal isolates used in this work have been deposited in the NCBI GenBank database under accession numbers PV664294–PV698418; individual accession numbers for each isolate are listed in **Table 1**. Further inquiries can be directed to the corresponding author.
