# Supplementary material for: Novel halotolerant fungal endophytes from hypersaline coastal ecosystems as sustainable biostimulants for early growth performance of tomato and wheat under saline stress
Source: Front Plant Sci. 2026 Aug 18;17:1905810. doi: 10.3389/fpls.2026.1905810 (PMC13529695; doi:10.3389/fpls.2026.1905810)
Supplement: Supplementary file 1 [file DataSheet1.docx]

Supplementary Material

**Table S1**. Radial growth rates (GR, cm d⁻¹) of fungal isolates grown on PDA medium amended with increasing NaCl concentrations (0, 2, 5, 10, 15, and 20 g L⁻¹) at three incubation temperatures (15, 25, and 35°C). Values represent the mean of five replicates ± standard deviation (SD). For each experimental condition, significant differences among isolates were determined by one-way ANOVA followed by Tukey's Honestly Significant Difference (HSD) post-hoc test (P < 0.05). Different lowercase superscript letters within each column indicate statistically significant differences between isolates.

| **Salinity al 15ºC (g L^-1^)** | | | | | | |
| --- | --- | --- | --- | --- | --- | --- |
| **Isolates** | **0** | **2** | **5** | **10** | **15** | **20** |
| **BC01** | 0.52±0.09def | 0.5±0cde | 0.33±0.03abcd | 0.37±0.04e | 0.25±0.00a | 0.22±0.01bc |
| **BC04** | 0.64±0.03fg | 0.54±0.04de | 0.82±0.04hijk | 0.7±0.04gh | 0.77±0.00i | 0.7±0.02i |
| **BC05** | 1.24±0.01l | 1.22±0.03k | 1.2±0.03lm | 1.23±0.01l | 1.19±0.05k | 1.24±0.01m |
| **BC06** | 1.17±0.14kl | 1.3±0.15jk | 1.32±0.14m | 1.2±0.05l | 1.18±0.01k | 1.14±0.00l |
| **BC08** | 0.6±0.1f | 0.85±0.35f | 0.58±0efg | 0.65±0.04g | 0.75±0.00i | 0.63±0.05h |
| **BC11** | 0.51±0.04def | 0.47±0.01cde | 0.48±0.01cde | 0.37±0.04e | 0.29±0.01abc | 0.28±0.01de |
| **BC12** | 0.83±0.2gh | 1.06±0.28hij | 1.05±0.03kl | 1.04±0.01k | 0.8±0.15i | 0.75±0.05ij |
| **BC13** | 0.49±0.04cdef | 0.38±0bcd | 0.39±0.03abcde | 0.33±0.04bcde | 0.27±0.03ab | 0.31±0.03e |
| **BC17** | 1.07±0.01jkl | 1.04±0.01hi | 0.98±0.01jkl | 0.77±0.01hi | 0.53±0.03ef | 0.46±0.03g |
| **BC18** | 1.06±0.05ijkl | 1.03±0.05ghi | 0.97±0.03jkl | 0.77±0.04hi | 0.59±0.05fg | 0.45±0.04fg |
| **BC20** | 1.13±0.02jkl | 1.01±0.01fghi | 0.9±0.04ijk | 0.95±0.18jk | 0.99±0.01j | 0.83±0.01k |
| **BC24** | 1.02±0.01hijk | 1.08±0.03ij | 1.43±0.66m | 0.83±0.13i | 0.78±0.0i | 0.79±0.03jk |
| **BC25** | 0.54±0.03ef | 0.6±0.07e | 0.61±0.11efgh | 0.63±0.06g | 0.73±0.15hi | 0.7±0.00i |
| **BC27** | 0.85±0.04ghi | 0.86±0.05fg | 0.83±0.03hijk | 0.79±0.1hi | 0.63±0.04fgh | 0.6±0.07h |
| **BC30** | 0.99±0.01hijk | 1.09±0.14ij | 0.99±0.05kl | 0.83±0.05i | 0.71±0.01hi | 0.59±0.06h |
| **BC31** | 0.16±0.01a | 0.18±0a | 0.29±0.08abcd | 0.22±0.03ab | 0.36±0.14bcd | 0.18±0b |
| **BC34** | 0.82±0.14gh | 0.97±0.06fghi | 0.92±0.06ijk | 0.88±0.16ij | 0.69±0.06ghi | 0.61±0.03h |
| **BC37** | 0.38±0.04bcde | 0.43±0.06cde | 0.42±0.01bcde | 0.33±0.05cde | 0.43±0.05de | 0.3±0.03e |
| **BC42** | 0.28±0.01abc | 0.25±0.05ab | 0.2±0.00ab | 0.23±0.0abcd | 0.2±0.05a | 0.23±0.06bcd |
| **BC44** | 0.27±0.05ab | 0.18±0a | 0.23±0.04ab | 0.24±0.01abcd | 0.23±0.01a | 0.23±0.03bcd |
| **BC45** | 0.33±0.07abcd | 0.35±0.13abc | 0.28±0.08abcd | 0.22±0.01abc | 0.21±0.03a | 0.21±0.04bc |
| **BC46** | 0.23±0.03ab | 0.23±0.03ab | 0.2±0.00ab | 0.2±0.02a | 0.2±0.00a | 0.25±0.07cde |
| **BC47** | 0.22±0.01ab | 0.23±0.04ab | 0.18±0.00a | 0.2±0.03a | 0.25±0a | 0.58±0.00h |
| **BC48** | 0.64±0.03fg | 0.5±0.13cde | 0.5±0.03def | 0.33±0.04de | 0.43±0.15de | 0.00±0.00a |
| **BC49** | 0.95±0.00hij | 0.9±0.03fgh | 0.75±0.00ghij | 0.6±0.11fg | 0.44±0.06de | 0.42±0.05fg |
| **BC50** | 0.68±0.04fg | 0.6±0.07e | 0.73±0.24fghi | 0.5±0.07f | 0.38±0.05cd | 0.40±0.00f |
| **BC51** | 0.18±0.01ab | 0.19±0.01a | 0.25±0.03abc | 0.28±0.03abcde | 0.25±0.04a | 0.23±0.05bcd |
| **BC52** | 0.21±0.01ab | 0.23±0.04ab | 0.33±0.17abcd | 0.24±0.01abcd | 0.25±0a | 0.23±0.05bcd |

**Table S1**. (Cont.)

| **Salinity at 25ºC (g L^-1^)** | | | | | | |
| --- | --- | --- | --- | --- | --- | --- |
| **Isolates** | **0** | **2** | **5** | **10** | **15** | **20** |
| **BC01** | 1.25±0.00efg | 1.03±0.18def | 0.89±0.01cd | 0.95±0.00ef | 0.81±0.08h | 0.68±0.15ef |
| **BC04** | 1.13±0.06def | 1.56±0.37ij | 1.55±0.52i | 1.17±0.10ghi | 1.04±0.18ij | 1.13±0.20ijk |
| **BC05** | 1.92±0.14j | 1.78±0.12k | 2.12±0.03k | 1.92±0.06l | 1.73±0.12n | 1.82±0.16m |
| **BC06** | 1.95±0.13j | 2.10±0.05l | 2.47±0.06l | 2.12±0.10m | 2.02±0.13o | 2.02±0.03n |
| **BC08** | 1.07±0.06d | 1.17±0.10efg | 1.20±0.00efg | 1.22±0.03hi | 1.22±0.03kl | 1.36±0.21l |
| **BC11** | 1.25±0.00efg | 1.20±0.09fgh | 1.00±0.22cde | 0.88±0.05e | 0.68±0.05g | 0.61±0.10def |
| **BC12** | 1.55±0.13hi | 1.62±0.10jk | 1.49±0.08hi | 1.39±0.01j | 1.15±0.04jk | 1.04±0.04hij |
| **BC13** | 1.19±0.10def | 1.15±0.13efg | 1.11±0.14ef | 0.87±0.04e | 0.69±0.01g | 0.63±0.04ef |
| **BC17** | 1.58±0.06hi | 1.73±0.03jk | 1.38±0.13ghi | 1.07±0.22fg | 0.86±0.13h | 0.66±0.09ef |
| **BC18** | 1.58±0.29hi | 1.67±0.03jk | 1.48±0.03hi | 1.17±0.14ghi | 0.83±0.04h | 0.76±0.01fg |
| **BC20** | 1.92±0.14j | 2.00±0.00l | 1.83±0.05j | 1.59±0.04k | 1.33±0.07lm | 1.23±0.15kl |
| **BC24** | 1.94±0.07j | 2.00±0.00l | 1.83±0.14j | 1.58±0.07k | 1.43±0.15m | 1.23±0.09kl |
| **BC25** | 1.12±0.01def | 1.11±0.03def | 1.18±0.08efg | 1.13±0.11gh | 1.13±0.05jk | 1.20±0.00jkl |
| **BC27** | 1.47±0.06h | 1.38±0.23hi | 1.32±0.12fgh | 1.25±0.00i | 0.99±0.00i | 0.90±0.05gh |
| **BC30** | 1.42±0.15gh | 1.33±0.08gh | 1.32±0.12fgh | 1.25±0.00i | 1.09±0.01ij | 0.93±0.03gh |
| **BC31** | 0.28±0.03a | 0.24±0.01a | 0.26±0.01a | 0.28±0.07a | 0.23±0.00a | 0.18±0.00a |
| **BC34** | 1.09±0.27de | 0.98±0.16de | 1.06±0.19de | 0.96±0.03ef | 0.98±0.03i | 1.01±0.04hi |
| **BC37** | 0.49±0.04bc | 0.50±0.00c | 0.55±0.03b | 0.52±0.03cd | 0.55±0.03f | 0.53±0.00cde |
| **BC42** | 0.33±0.03ab | 0.30±0.03abc | 0.33±0.00a | 0.37±0.01ab | 0.29±0.01abc | 0.25±0.03ab |
| **BC44** | 0.30±0.03a | 0.29±0.05ab | 0.29±0.04a | 0.28±0.03a | 0.25±0.00ab | 0.28±0.03ab |
| **BC45** | 0.53±0.09c | 0.48±0.04bc | 0.43±0.03ab | 0.42±0.04bc | 0.44±0.01cd | 0.41±0.01bc |
| **BC46** | 0.35±0.03ab | 0.33±0.01abc | 0.28±0.01a | 0.26±0.04a | 0.26±0.02abc | 0.26±0.01ab |
| **BC47** | 0.34±0.04ab | 0.33±0.03abc | 0.26±0.01a | 0.29±0.01a | 0.28±0.00abc | 0.25±0.00ab |
| **BC48** | 1.27±0.04fg | 0.93±0.05d | 0.79±0.07c | 0.53±0.10cd | 0.49±0.01ef | 0.4±0.00bc |
| **BC49** | 1.68±0.03i | 1.79±0.30k | 1.49±0.01hi | 1.64±0.10k | 1.38±0.05m | 1.16±0.39ijk |
| **BC50** | 1.24±0.01defg | 1.03±0.14def | 0.81±0.12c | 0.58±0.06d | 0.35±0.03bcd | 0.43±0.00bcd |
| **BC51** | 0.28±0.03a | 0.33±0.04abc | 0.30±0.00a | 0.30±0.03ab | 0.33±0.00abcd | 0.28±0.00ab |
| **BC52** | 0.39±0.14abc | 0.28±0.00a | 0.29±0.04a | 0.26±0.01a | 0.36±0.04cd | 0.21±0.01a |

**Table S1**. (Cont.)

| **Salinity at 35ºC (g L^-1^)** | | | | | | |
| --- | --- | --- | --- | --- | --- | --- |
| **Isolates** | **0** | **2** | **5** | **10** | **15** | **20** |
| **BC01** | 1.00±0.13f | 0.86±0.01d | 0.88±0.00c | 0.84±0.01b | 0.73±0.01d | 0.63±0.00f |
| **BC04** | 1.21±0.04g | 1.37±0.10g | 1.53±0.43e | 1.42±0.20c | 1.15±0.09f | 1.19±0.01h |
| **BC05** | 1.18±0.07g | 1.92±0.06h | 2.23±0.03gh | 2.17±0.15d | 2.18±0.20h | 2.17±0.14j |
| **BC06** | 1.24±0.01g | 2.17±0.20i | 2.42±0.03h | 2.45±0.05e | 2.40±0.00i | 2.42±0.03k |
| **BC08** | 1.25±0.00g | 1.08±0.18e | 1.23±0.04d | 1.28±0.24c | 1.20±0.00fg | 1.22±0.03h |
| **BC11** | 0.91±0.01e | 1.19±0.06f | 1.16±0.47d | 0.98±0.04b | 0.71±0.05cd | 0.79±0.08g |
| **BC12** | 0.10±0.02bc | 0.14±0.01bc | 0.13±0.00ab | 0.15±0.00a | 0.15±0.00b | 0.13±0.00bc |
| **BC13** | 0.88±0.00e | 0.91±0.09d | 0.90±0.03c | 0.84±0.01b | 0.68±0.05cd | 0.63±0.01f |
| **BC17** | 0.14±0.01bcd | 0.19±0.01bcb | 0.17±0.03ab | 0.13±0.06a | 0.15±0.00b | 0.12±0.01bc |
| **BC18** | 0.10±0.02bc | 0.09±0.04abc | 0.13±0.01ab | 0.10±0.00a | 0.11±0.00b | 0.13±0.00bc |
| **BC20** | 0.09±0.01bc | 0.09±0.01abc | 0.10±0.00ab | 0.13±0.00a | 0.12±0.01b | 0.12±0.01bc |
| **BC24** | 0.00±0.00a | 0.00±0.00a | 0.00±0.00a | 0.00±0.00a | 0.00±0.00a | 0.00±0.00a |
| **BC25** | 1.19±0.01g | 1.23±0.03f | 1.29±0.14d | 1.42±0.33c | 1.30±0.18g | 1.67±0.12i |
| **BC27** | 0.17±0.16cd | 0.10±0.03abc | 0.24±0.31 | 0.11±0.01a | 0.12±0.01b | 0.13±0.00bc |
| **BC30** | 0.09±0.01b | 0.08±0.01ab | 0.10±0.01ab | 0.13±0.00a | 0.13±0.00b | 0.10±0.00b |
| **BC31** | 0.00±0.00a | 0.00±0.00a | 0.00±0.00a | 0.00±0.00a | 0.00±0.00a | 0.00±0.00a |
| **BC34** | 0.00±0.00a | 0.00±0.00a | 0.00±0.00a | 0.00±0.00a | 0.00±0.00a | 0.00±0.00a |
| **BC37** | 0.00±0.00a | 0.00±0.00a | 0.00±0.00a | 0.00±0.00a | 0.00±0.00a | 0.00±0.00a |
| **BC42** | 0.13±0.01bcd | 0.13±0.01bc | 0.13±0.00ab | 0.18±0.03a | 0.16±0.01b | 0.13±0.01bc |
| **BC44** | 0.18±0.01d | 0.16±0.01bc | 0.14±0.01ab | 0.15±0.03a | 0.17±0.01b | 0.19±0.04cd |
| **BC45** | 0.16±0.04bcd | 0.14±0.01bc | 0.09±0.01ab | 0.13±0.04a | 0.13±0.00b | 0.12±0.01bc |
| **BC46** | 0.17±0.01bcd | 0.18±0.06bc | 0.17±0.03ab | 0.14±0.01a | 0.16±0.01b | 0.22±0.06d |
| **BC47** | 0.19±0.01d | 0.19±0.01c | 0.20±0.03abb | 0.18±0.03a | 0.17±0.01b | 0.17±0.01bcd |
| **BC48** | 2.47±0.12j | 2.34±0.08j | 1.90±0.03f | 1.29±0.35c | 0.69±0.09cd | 0.60±0.03f |
| **BC49** | 1.93±0.05h | 1.98±0.00h | 1.83±0.01f | 1.5±0.07c | 1.01±0.12e | 0.43±0.03e |
| **BC50** | 2.28±0.05i | 2.38±0.07j | 2.06±0.08fg | 1.36±0.39c | 0.63±0.00c | 0.46±0.01e |
| **BC51** | 0.19±0.03d | 0.15±0.03bc | 0.14±0.04ab | 0.13±0.06a | 0.15±0.03b | 0.17±0.01bcd |
| **BC52** | 0.14±0.01bcd | 0.14±0.01bc | 0.16±0.04ab | 0.14±0.01a | 0.13±0.00b | 0.15±0.00bcd |

**Table S2.** Effect of fungal inoculation on seed germination in tomato (*Solanum lycopersicum* L.) under increasing NaCl concentrations. Values represent the mean number of germinated seeds (± SD) out of 12 seeds per replicate (*n* = 3). C: non-inoculated control. Different lowercase superscript letters within each column indicate statistically significant differences among isolates (Tukey's HSD, *p* < 0.05). *p*-values from one-way ANOVA for each salinity level are shown in the last row.

|  | **Salinity (g L^-1^)** | | | | |
| --- | --- | --- | --- | --- | --- |
| **Isolates** | **0** | **25** | **50** | **75** | **100** |
| **BC01** | 11.33±0.58a | 10.33±0.58ab | 9.67±0.58a | 7.00±0.00a | 6.33±0.58a |
| **BC04** | 11.33±0.58a | 10.00±1.00ab | 11.67±0.58a | 10.33±1.15ab | 9.33±0.58cdef |
| **BC05** | 11.67±0.58a | 11.00±0.00b | 9.67±1.53a | 11.33±1.15b | 9.00±1.73bcdef |
| **BC06** | 11.00±1.00a | 11.67±0.25b | 11.67±0.58a | 12.00±0.00b | 12.00±0.00h |
| **BC08** | 9.33±1.53a | 10.67±0.58b | 11.33±1.15a | 9.33±2.08ab | 9.67±0.58cdefg |
| **BC11** | 10.33±0.58a | 10.67±0.58b | 9.67±2.31a | 11.33±1.15b | 8.67±2.08bcde |
| **BC12** | 10.67±1.53a | 10.67±0.58b | 10.00±1.00a | 9.00±2.00ab | 9.00±2.65bcdef |
| **BC13** | 10.67±0.58a | 11.33±0.58b | 10.00±2.00a | 9.33±0.58ab | 7.00±1.73ab |
| **BC17** | 11.33±0.58a | 11.00±1.00b | 10.33±0.58a | 10.33±0.58ab | 11.67±0.58gh |
| **BC18** | 11.33±0.58a | 11.67±0.58b | 10.00±0.00a | 10.67±1.53ab | 10.00±1.00cdefgh |
| **BC20** | 11.67±0.58a | 11.33±0.58b | 11.67±0.58a | 9.33±1.53ab | 9.33±2.08cdef |
| **BC24** | 11.00±1.00a | 10.00±0.00ab | 10.67±1.53a | 10.33±1.15ab | 10.00±1.00cdefgh |
| **BC25** | 10.33±1.15a | 11.00±1.00b | 11.67±0.58a | 10.67±0.25ab | 9.00±1.00bcdef |
| **BC27** | 12.00±0.00a | 11.00±1.00b | 11.00±1.00a | 11.00±1.00b | 9.33±2.52cdef |
| **BC30** | 11.33±1.15a | 11.33±0.58b | 11.67±0.58a | 9.00±0.00ab | 10.33±1.15defgh |
| **BC31** | 10.67±0.58a | 11.00±1.73b | 10.67±1.53a | 10.33±0.58ab | 10.00±2.00cdefgh |
| **BC34** | 9.67±1.15a | 9.00±1.73ab | 10.33±0.58a | 11.00±1.00b | 10.33±0.58defgh |
| **BC37** | 11.33±0.58a | 10.33±1.15ab | 11.67±0.58a | 11.67±0.58b | 10.00±1.73cdefgh |
| **BC42** | 10.33±1.15a | 10.67±1.53b | 9.67±0.58a | 10.67±0.58ab | 9.67±1.53cdefg |
| **BC44** | 11.67±0.58a | 11.67±0.58b | 12.00±0.00a | 10.67±1.53ab | 10.67±0.58efgh |
| **BC45** | 11.33±0.58a | 11.00±1.00b | 10.67±0.58a | 11.00±1.73b | 8.67±1.15bcde |
| **BC46** | 11.33±0.58a | 10.33±0.58ab | 11.00±0.00a | 11.67±0.58b | 9.67±1.53cdefg |
| **BC47** | 10.00±1.00a | 9.67±0.58ab | 10.33±0.58a | 10.00±2.00ab | 11.00±0.00fgh |
| **BC48** | 11.00±0.00a | 10.67±1.53b | 11.33±0.58a | 11.00±1.00b | 8.00±1.00abc |
| **BC49** | 11.67±0.58a | 10.67±0.58b | 10.67±1.53a | 9.33±1.53ab | 8.33±1.53abcd |
| **BC50** | 11.33±0.58a | 10.33±1.15ab | 9.67±0.58a | 10.33±0.58ab | 8.33±0.58abcd |
| **BC51** | 10.67±1.53a | 8.00±1.00a | 11.00±0.00a | 11.00±1.00b | 9.33±2.08cdef |
| **BC52** | 10.00±1.00a | 11.33±0.58b | 11.00±1.00a | 11.67±0.58b | 11.66±0.58gh |
| **C** | 11.00±1.00a | 11.33±0.58b | 12.00±0.00a | 11.00±1.00b | 9.33±1.15cdef |
| ***p-value*** | *0.0365* | *0.0011* | *0.0176* | *0.0011* | *0.0019* |

**Table S3.** Effect of fungal inoculation on seed germination in wheat (*Triticum aestivum* L.) under increasing NaCl concentrations. Values represent the mean number of germinated seeds (± SD) out of 16 seeds per replicate (*n* = 3). C: non-inoculated control. Different lowercase superscript letters within each column indicate statistically significant differences among isolates (Tukey's HSD, *p*< 0.05). *p*-values from one-way ANOVA for each salinity level are shown in the last row.

|  | | **Salinity (g L^-1^)** | | | | | | | | | |
| --- | --- | --- | --- | --- | --- | --- | --- | --- | --- | --- | --- |
| **Isolates** | | **0** | **50** | **75** | | **100** | **150** | | **200** | **250** | **300** |
| **BC01** | 11.67±1.15abc | | 13.67±0.58ab | 13.67±0.58abc | 13.33±0.58ab | | 7.67±0.58abcd | 8.00±0.00defghi | | 4.33±1.53efgh | 0.00±0.00a |
| **BC04** | 13.00±1.00bcd | | 14.00±0.00ab | 14.67±0.58bc | 13.33±2.08ab | | 4.33±1.53a | 4.00±1.00ab | | 5.67±0.58ghi | 0.00±0.00a |
| **BC05** | 14.67±0.58cd | | 13.67±0.58ab | 13.33±1.53abc | 13.33±2.08ab | | 11.33±1.15efghij | 10.33±0.58ij | | 10.33±0.58j | 2.00±1.15c |
| **BC06** | 11.33±0.58ab | | 13.00±1.00ab | 13.33±1.53abc | 11.67±0.58ab | | 11.33±1.53efghij | 9.00±1.00fghij | | 8.00±1.00ij | 5.33±1.15d |
| **BC08** | 13.33±1.15bcd | | 12.67±2.08ab | 13.33±1.15abc | 12.67±0.58ab | | 7.67±0.58abcd | 8.00±0.00defghi | | 7.33±0.58i | 0.00±0.00a |
| **BC11** | 14.00±0.00bcd | | 13.67±1.53ab | 13.00±1.00abc | 14.67±0.58b | | 9.00±0.00cdefg | 4.33±0.58ab | | 1.33±0.58abc | 0.00±0.00a |
| **BC12** | 11.67±2.08abc | | 14.00±1.00ab | 12.33±0.58abc | 12.00±1.00ab | | 13.67±0.58j | 6.33±1.15bcdef | | 1.67±0.58abcd | 0.33±0.58ab |
| **BC13** | 14.33±0.58bcd | | 13.33±0.58ab | 13.00±0.00abc | 11.67±0.58ab | | 7.00±1.00abc | 5.67±1.15abcde | | 4.67±0.58fgh | 0.33±0.58ab |
| **BC17** | 15.00±1.00d | | 14.00±0.00ab | 14.33±0.58bc | 13.00±1.00ab | | 10.00±1.00cdefghi | 4.67±0.58abc | | 1.33±0.58abc | 0.00±0.00a |
| **BC18** | 15.33±0.58d | | 15.00±0.00ab | 14.00±0.00abc | 13.00±2.00ab | | 9.33±1.15cdefgh | 9.67±0.58ghij | | 6.00±1.00hi | 0.00±0.00a |
| **BC20** | 13.67±2.08bcd | | 13.33±1.15ab | 12.67±1.53abc | 15.00±1.73b | | 8.00±1.00bcde | 11.33±1.53j | | 3.67±0.58cdefgh | 0.00±0.00a |
| **BC24** | 13.33±2.08bcd | | 13.67±2.52ab | 12.67±1.15abc | 12.67±1.15ab | | 9.33±1.15cdefgh | 10.00±0.00hij | | 3.67±0.58cdefgh | 0.00±0.00a |
| **BC25** | 14.33±0.58bcd | | 15.67±0.58b | 13.33±2.31abc | 12.00±0.00ab | | 11.00±1.00defghij | 7.00±0.00bcdefgh | | 5.00±0.58fgh | 0.00±0.00a |
| **BC27** | 15.00±1.00d | | 13.00±1.00ab | 13.67±1.15abc | 15.33±1.15b | | 7.33±1.53abc | 5.00±2.00abcd | | 2.00±1.00abcde | 1.33±0.58bc |
| **BC30** | 14.00±0.00bcd | | 14.33±0.58ab | 12.67±1.53abc | 14.00±1.73ab | | 12.33±1.15ghij | 6.67±1.53bcdefg | | 1.00±1.00ab | 0.33±0.58ab |
| **BC31** | 14.33±0.58bcd | | 13.00±1.00ab | 10.00±1.73a | 14.33±1.15b | | 13.67±1.15j | 11.33±0.58j | | 4.67±1.15fgh | 0.00±0.00a |
| **BC34** | 15.33±0.58d | | 13.33±0.58ab | 13.00±1.73abc | 13.67±1.53ab | | 11.67±1.15fghij | 3.00±1.00a | | 0.67±0.58a | 0.33±0.58ab |
| **BC37** | 14.67±0.58cd | | 14.00±1.00ab | 14.67±2.31bc | 12.33±1.15ab | | 13.67±0.58j | 10.00±1.00hij | | 1.00±0.00ab | 0.00±0.00a |
| **BC42** | 15.00±1.00d | | 14.33±0.58ab | 12.00±1.73abc | 15.00±1.00b | | 8.67±0.58bcdef | 8.67±0.58efghij | | 2.00±1.00abcde | 0.33±0.58ab |
| **BC44** | 15.00±1.00d | | 14.67±0.58ab | 14.33±1.15bc | 13.00±1.00ab | | 13.67±0.58j | 7.67±0.58cdefghi | | 4.00±1.00defgh | 1.00±0.00abc |
| **BC45** | 13.00±1.00bcd | | 15.67±0.58b | 12.33±1.15abc | 13.00±1.00ab | | 13.00±1.00ij | 10.33±2.08ij | | 3.33±0.58bcdefg | 0.00±0.00a |
| **BC46** | 14.67±0.58cd | | 13.67±1.15ab | 13.33±0.58abc | 12.67±0.58ab | | 14.00±2.00j | 8.00±1.73defghi | | 4.33±1.53efgh | 0.00±0.00a |
| **BC47** | 14.00±1.00bcd | | 15.67±0.58b | 15.33±0.58c | 14.67±0.58b | | 12.33±0.58ghij | 10.00±1.00hij | | 3.33±0.58bcdefg | 0.33±0.58ab |
| **BC48** | 14.67±0.58cd | | 13.33±1.53ab | 12.33±0.58ab | 15.00±0.00b | | 12.67±1.15hij | 10.33±0.58ij | | 0.67±0.58a | 0.67±0.58ab |
| **BC49** | 14.33±0.58bcd | | 11.33±1.53a | 14.33±1.15bc | 13.00±0.00ab | | 14.33±0.58j | 5.67±1.53abcde | | 2.00±1.00abcde | 0.33±0.58ab |
| **BC50** | 14.67±0.58cd | | 14.33±1.53ab | 14.00±1.00abc | 13.33±0.58ab | | 13.00±1.00ij | 10.00±0.00hij | | 1.33±0.58abc | 0.00±0.00a |
| **BC51** | 15.33±0.58d | | 14.33±1.15ab | 13.33±1.15abc | 13.67±1.53ab | | 7.33±1.53abc | 5.67±1.15abcde | | 3.00±1.15abcdef | 0.00±0.00a |
| **BC52** | 14.33±0.58bcd | | 13.67±1.53ab | 13.00±2.00abc | 13.67±1.53ab | | 13.67±1.53j | 6.67±1.15bcdefg | | 3.33±0.58bcdefg | 0.00±0.00a |
| **C** | 9.33±2.60a | | 11.00±1.00a | 11.33±0.58ab | 10.33±0.58a | | 5.33±1.15ab | 3.00±0.00a | | 2.00±0.00abcde | 0.00±0.00a |
| ***p-value*** | *0.0000* | | *0.0253* | *0.0062* | *0.0002* | | *0.0000* | *0.0000* | | *0.0000* | *0.0000* |

**Table S4.** Effect of fungal inoculation on shoot dry weight, root dry weight and total biomass of tomato (*Solanum lycopersicum* L.) seedlings grown under non-saline (N) and saline (S, 100 mM NaCl) conditions. Values represent the mean ± standard deviation (n = 20). C: non-inoculated control. Different lowercase superscript letters within each column indicate statistically significant differences among treatments (Tukey’s HSD, p < 0.05).

|  | **Shoot dry weight (g)** |  | **Root dry weight (g)** |  | **Total biomass (g)** |  |
| --- | --- | --- | --- | --- | --- | --- |
| **Isolate** | **N** | **S** | **N** | **S** | **N** | **S** |
| BC01 | 0.192±0.119f | 0.260±0.030abcdefgh | 0.051±0.037f | 0.046±0.005cde | 0.242±0.154i | 0.307±0.032abcdefg |
| BC04 | 0.877±0.168ab | 0.377±0.169abcdefg | 0.122±0.061cdef | 0.055±0.040cde | 0.999±0.217abc | 0.432±0.206abcdef |
| BC05 | 0.607±0.199abcde | 0.508±0.118ab | 0.197±0.066cd | 0.112±0.041bc | 0.804±0.256bcdef | 0.620±0.149ab |
| BC06 | 0.773±0.205abc | 0.495±0.198ab | 0.211±0.061bc | 0.142±0.060b | 0.984±0.259abcd | 0.637±0.254b |
| BC08 | 0.701±0.254abcd | 0.140±0.062efgh | 0.180±0.101cde | 0.039±0.007cde | 0.881±0.346abcde | 0.179±0.063efg |
| BC11 | 0.240±0.087ef | 0.052±0.031h | 0.114±0.028cdef | 0.011±0.013e | 0.354±0.072efghi | 0.063±0.041g |
| BC12 | 0.194±0.164f | 0.137±0.087defgh | 0.020±0.013f | 0.022±0.011de | 0.215±0.156i | 0.159±0.097efg |
| BC13 | 0.256±0.139ef | 0.256±0.054abcdefgh | 0.069±0.028ef | 0.072±0.021bcde | 0.325±0.161ghi | 0.328±0.074abcdefg |
| BC17 | 0.361±0.149def | 0.438±0.149abcd | 0.069±0.028ef | 0.095±0.037bcd | 0.430±0.167efghi | 0.533±0.181abcd |
| BC18 | 0.252±0.142ef | 0.370±0.089abcdefg | 0.041±0.033f | 0.081±0.039bcde | 0.293±0.175hi | 0.452±0.125abcdef |
| BC20 | 0.466±0.173cdef | 0.198±0.056cdefgh | 0.055±0.023ef | 0.028±0.008de | 0.521±0.189cdefghi | 0.226±0.062defg |
| BC24 | 0.397±0.199cdef | 0.100±0.071fgh | 0.102±0.068cdef | 0.006±0.006e | 0.499±0.222defghi | 0.105±0.076fg |
| BC25 | 0.221±0.065ef | 0.191±0.138defgh | 0.045±0.030f | 0.047±0.035cde | 0.266±0.091hi | 0.237±0.170defg |
| BC27 | 0.326±0.163def | 0.205±0.048cdefgh | 0.065±0.028ef | 0.047±0.025cde | 0.392±0.185fghi | 0.252±0.063cdefg |
| BC30 | 0.619±0.164abcde | 0.401±0.043abcdefg | 0.132±0.032cdef | 0.046±0.011cde | 0.751±0.190bcdefgh | 0.447±0.042abcdef |
| BC31 | 0.393±0.253cdef | 0.120±0.102gh | 0.092±0.059cdef | 0.015±0.011e | 0.485±0.310defghi | 0.136±0.107fg |
| BC34 | 0.528±0.186bcdef | 0.465±0.093abcd | 0.144±0.048cdef | 0.146±0.041b | 0.672±0.228bcdefghi | 0.611±0.133abc |
| BC37 | 0.438±0.085cdef | 0.195±0.060cdefgh | 0.082±0.015cdef | 0.050±0.026cde | 0.519±0.090cdefghi | 0.245±0.081defg |
| BC42 | 0.249±0.129ef | 0.221±0.071bcdefgh | 0.047±0.026f | 0.253±0.060a | 0.296±0.152ghi | 0.474±0.109abcdef |
| BC44 | 0.462±0.238cdef | 0.467±0.108abc | 0.209±0.114c | 0.117±0.029bc | 0.671±0.301bcdefghi | 0.584±0.118abc |
| BC45 | 0.975±0.100a | 0.404±0.330abcdefg | 0.340±0.059ab | 0.092±0.071bcde | 1.315±0.138a | 0.496±0.395abcde |
| BC46 | 0.794±0.468abc | 0.539±0.131a | 0.347±0.138a | 0.101±0.038bcd | 1.141±0.571ab | 0.641±0.166ab |
| BC47 | 0.526±0.085bcdef | 0.295±0.040abcdefgh | 0.105±0.031cdef | 0.079±0.026bcde | 0.631±0.108bcdefghi | 0.374±0.061abcdefg |
| BC48 | 0.522±0.112bcdef | 0.252±0.086abcdefgh | 0.102±0.031cdef | 0.050±0.029cde | 0.625±0.140cdefghi | 0.302±0.114acdefg |
| BC49 | 0.590±0.188abcde | 0.406±0.206abcde | 0.194±0.062cd | 0.107±0.039bc | 0.784±0.244bcdefg | 0.513±0.195abcde |
| BC50 | 0.543±0.112bcdef | 0.447±0.135abcd | 0.121±0.072cdef | 0.097±0.011bcd | 0.663±0.149bcdefghi | 0.544±0.144abcd |
| BC51 | 0.252±0.100ef | 0.415±0.088abcde | 0.058±0.040ef | 0.092±0.028bcd | 0.310±0.136ghi | 0.507±0.099abcde |
| BC52 | 0.481±0.069cdef | 0.405±0.112abcdef | 0.144±0.021cdef | 0.098±0.035bcd | 0.625±0.085cdefghi | 0.502±0.141abcde |
| **C** | **0.469±0.126cdef** | **0.334±0.123abcdefgh** | **0.077±0.035def** | **0.057±0.026cde** | **0.546±0.110cdefghi** | **0.392±0.111abcdefg** |

**Supplementary Figure S1.** Growth-chamber setup of the experimental assays. **(A)** Tomato (*Solanum lycopersicum*) plants grown in trays under controlled conditions, photographed 25 days after transplanting. **(B)** Wheat (*Triticum aestivum*) germination trays under the same controlled conditions, photographed at the beginning of the assay (approximately 10 days). Trays correspond to the different treatments described in the Materials and Methods; treatments were coded to keep the identity of the fungus concealed during the assay and thus avoid biased assessment.

**Supplementary Figure S2.** Salt-buffering effect of *Sordaria fimicola* on tomato plants. **(A)** Control, 0 g L⁻¹ salinity; **(B)** control, 100 g L⁻¹ salinity; **(C)** *S. fimicola*, 0 g L⁻¹ salinity; **(D)** *S. fimicola*, 100 g L⁻¹ salinity. In non-inoculated plants, salinity reduced plant vigour (compare **A** and **B**), whereas in plants inoculated with *S. fimicola* growth was maintained under salt stress (compare **C** and **D**), indicating a buffering of the salt effect in the presence of the fungus. Plants were photographed after 25 days of treatment.

**Supplementary Figure S3.** Shoot and root development of wheat (*Triticum aestivum*) inoculated with fungal isolates that showed a statistically significant effect on plant growth relative to the uninoculated control, under 150 mM salinity, including isolates that significantly enhanced growth and those that reduced it. Plants were removed from the substrate and photographed after 56 days.

**Supplementary Figure S4.** Confirmation of endophytic colonization by inoculated fungal strains**.** **(A–E)** Re-isolation of the inoculated fungal strains from surface-sterilized root fragments on PDA (Potato Dextrose Agar), confirming their internal (endophytic) colonization of the root system: (A) Several DSE isolates belonging to *Clarireedia*; (B) *Torula* sp., (C) *Macrophomina phaseolina*; (D) *Sordaria fimicola* and *Trichoderma gamsii*; (E) *Torula* sp. and *T. saturnisporum*; **(F–J)** Microscopic visualization of fungal structures colonizing root tissue in the corresponding isolates: (F) *C. narcisi*; (G) Cleistothecia of *S. fimicola*; (H) *Macrophomina phaseolina* (I) *Torula* sp. (J) *N. gossypiicola*.

.

**Supplementary Figure S5.** Light micrographs of the radicle of germinated tomato and wheat seeds colonised by fungal species. (A) Tomato radicle with *Sordaria fimicola*; (B) tomato radicle with *Clarireedia narcissi*; (C) wheat radicle with *Sordaria fimicola*. Fungal hyphae are visible growing over the radicle surface. Scale bars = 1000 µm.
